# Acidity and availability of aluminum, iron and manganese as factors affecting germination in European acidic dry and alkaline xerothermic grasslands

**DOI:** 10.7717/peerj.13255

**Published:** 2022-04-28

**Authors:** Mateusz Wala, Jeremi Kołodziejek, Tomasz Wilk

**Affiliations:** 1 Department of Geobotany and Plant Ecology, Faculty of Biology and Environmental Protection, University of Lodz, Łódź, Łódź Voivodeship, Poland; 2 Przedsiębiorstwo Produkcyjno-Consultingowe ADOB Sp. z o.o. Sp. jawna, Poznań, Greater Poland Voivodeship, Poland; 3 Faculty of Chemistry, Adam Mickiewicz University in Poznań, Poznań, Greater Poland Voivodeship, Poland

**Keywords:** Germination, Iron, Manganese, Aluminum, pH, Grasslands

## Abstract

Germination ecology of 10 species from acidic dry grasslands and 10 species from alkaline xerothermic grasslands was studied. The seeds were subjected to different pH, iron (Fe), manganese (Mn) and aluminum (Al) treatments under controlled conditions. Effects of ionic (chlorides) and chelated forms (HBED chelates) of Fe and Mn were also compared. Final germination percentage (FGP) and index of germination velocity (IGV) were calculated. The results indicate that pH and extremely high availability of Al are the major edaphic filters regulating germination-based revegetation, while availability of Fe and Mn is of the secondary importance. Both chelates and ionic forms of Fe and Mn exerted similar effects on the ability of seeds to complete germination. It suggests that both chelates are not hazardous for early ontogenetic stages of plants. Neither group has group-specific adaptations pertaining to germination characteristics in the context of the studied chemical stimuli, which indicates a diversity of germination strategies and individual species-specific reactions to the tested factors.

## Introduction

Germination is a process which is controlled by environmental conditions (Koornneef, Bentsink & Hilhorst, 2002). Light, temperature and availability of water are known to be crucial factors affecting completion of germination of seeds (Gibson, 2009; Graeber et al., 2012). However, there are also many secondary (but important) abiotic factors affecting germination, such as availability and type of nitrogen-containing compounds and other macronutrients, acidity (pH), availability of micronutrients and nonessential trace/ballast elements as well as the amount and type of pollutants (Graeber et al., 2012; Kranner & Colville, 2011). Therefore, completion of germination that is observed in the field depends on a multifactorial interaction between environmental determinants. These stimuli directly affect metabolism of seeds and/or they are recognized and translated into a signal affecting hormonal equilibrium that prevents or triggers germination (Nonogaki, 2017; Duermeyer et al., 2018).

Grasslands are plant communities characterized with high solar irradiance and light heterogeneity (Bakker, Blair & Knapp, 2003). Light that reaches the soil surface of ‘vegetation gaps’, where completion of germination is most favored, is not strongly filtered due to the lack of dense canopy. Thus, it has an optimal spectrum that supports the germination process of photoblastic-positive plant species (Deregibus et al., 1985). Temperature of soil surface, where seeds of photoblastic-positive plant species complete germination, depends partially on physical-chemical properties of the soil (Bothe, 2015). Although non-calcareous sandy soils and calcareous soils can reach, respectively, 70 °C and 60 °C on a sunny day in summer (Jentsch & Beyschlag, 2003; Bothe, 2015), the temperatures of soil surface and diurnal amplitude of soil temperature in spring and autumn (when seeds of many herbs complete germination; Czarnecka, 2004) are lesser (Burmeier et al., 2010). This creates a safe environment in terms of major factors controlling the germination process. Soil solution, which makes imbibition of seeds possible, contains a wide spectrum of chemical compounds (Marques et al., 1996). Many of them interfere with the germination process, enhancing (*e.g.*, the appropriate form and dose of nitrogen; Duermeyer et al., 2018) or halting it (*e.g.*, due to toxicity of ions or due to supraoptimal osmoticum; Kranner & Colville, 2011).

Dry acidic grasslands from *Koelerio-Corynephoretea* Klika in Klika et V. Novák 1941 class and xerothermic alkaline grasslands from *Festuco-Brometea* Br.-Bl. et R.Tx. 1943 class are the priority types of habitats in Europe (*6120 and *6210, respectively; Council Directive, 1992). Plants from those habitats share two common traits, namely, tolerance to heat stress and high irradiance (Leuchner & Ellenberg, 2017). However, there are several iconic characteristics of each type of soil that differentiate between them (Lee, 1999; Bothe, 2015). Soils of dry acidic grasslands (mostly Podzols) are known to be remarkably acidic and to contain high concentrations of available Al, Mn and Fe due to low pH (Abedi, Bartelheimer & Poschlod, 2013; Strawn, Bohn & O’Connor, 2020). Depending on many soil-forming factors, concentration of Ca in non-calcareous sandy soils vary, but it is established that typical inland sandy soils (not affected by marine or alluvial deposition) are poor in this element (Török et al., 2009). Soils on which alkaline xerothermic grasslands develop (mostly Rendzinas) are completely opposite—they are rich in Ca and are characterized with low acidity due to composition of bedrock (in most cases bedrock is rich in CaCO_3_; sometimes it originates from CaSO_4_—then they are susceptible to acidification). Furthermore, due to high pH, several elements, *e.g.*, Fe, Mn and Al are precipitated in insoluble forms (Bothe, 2015; Strawn, Bohn & O’Connor, 2020). Considering two major characteristics of alkaline and acidic soils, namely availability of Ca and soil pH, plants on calcareous soils are recognized as calcicoles (sometimes referred to as basophiles or calciphiles), whereas those on silicate soils are calcifuges (or calciphobes/acidophiles, considering their reaction to Ca and pH; Bothe, 2015). Interestingly, the abovementioned factors (pH and availability of Ca, Fe, Mn and Al) were only rarely studied in the past as the factors affecting germination process in those types of grasslands.

It is known that different forms of mineral nutrients and ballast elements are not equally available for plants. For example, availability of Al is driven by soil pH, as soil solutions of acidic soils are remarkably rich in this element, while in alkaline soils its availability is definitely lesser (Abedi, Bartelheimer & Poschlod, 2013; Bothe, 2015). In the case of Fe and Mn, the most common species available for plants are their free ions (strictly limited source) and complexed forms (Bothe, 2015; Shao et al., 2017). Relatively much is known of Fe and Mn nutrition and effects of availability of these two elements on vegetative growth of higher plants (Andresen, Peiter & Küpper, 2018). Chelated Fe can be readily acquired and utilized (Strategy II plants) or reduced and then acquired by plants (Strategy I plants; Grillet & Schmidt, 2017). Furthermore, Fe and Mn are subjected to constant ligand (chelator) exchange (including competition of these metals for a given chelator), which probably shifts their availability in soil environment (Yehuda et al., 1996; Duckworth, Bargar & Sposito, 2009). The phenomenon of metal chelation (by biological as well as synthetic chelators, *e.g.*, *N*,*N*’-di(2-hydroxybenzyl)ethylenediamine-*N*,*N*’-diacetic acid; HBED) underlies research on soil chemistry and nutrition of plants (where chelates can be used as a tool; Strawn, Bohn & O’Connor, 2020; Wala et al., 2020), but it was also found to be a remedy for losses of productivity in agriculture (Venturas et al., 2014). Even apart from the scientific and economical significance of this problem, there are some other practical issues associated with the influence of ionic and chelated forms of Fe and Mn on plants. Although the usage of metal chelates in agriculture and horticulture has gained popularity in recent years, not much is known about their environmental effects and safety, including their influence on the early ontogenetic stages of plants. According to the available data, HBED chelates were not studied in this context. Furthermore, Mn-HBED was never investigated in the context of plant physiology, thus nothing is known about its mode of action and safety.

**Table 1:** List of the studied plant species, their growth forms, seed characteristics, habitat preferences and ecological indicator values describing their realized centers of abundance.

Species	Abbreviation	Family	Growth Form^a^	Seed size (mm)^b^	Seed weight (mg)^c^	Preference to soil pH^d^	Ellenberg’s indicator value^e^					
							L	T	K	F	R	N
*Alyssum montanum* L.	Amo	Brassicaceae	P	1.5–1.9 × 1.1–1.3	0.369 ± 0.002	A	9	6	4	2	7	1
*Aster amellus* L.	Aam	Asteraceae	P	3.4–3.8 × 1.5–1.8	1.010 ± 0.029	B	8	6	6	4	9	3
*Betonica officinalis* L.	Bof	Lamiaceae	P	2.7–3.1 × 1.3–1.5	1.147 ± 0.035	B	7	6	5	*	0	3
*Centaurea scabiosa* L.	Csc	Asteraceae	P	4.5–5.0 × 2.0–2.2	4.815 ± 0.211	B	7	0	3	3	8	4
*Centaurea stoebe* Tausch	Cst	Asteraceae	B	2.5–3.0 × 1.2–1.4	2.146 ± 0.033	A	8	7	5	2	8	3
*Dianthus carthusianorum* L.	Dca	Caryophyllaceae	P	2.0–2.4 × 1.5–1.8	0.283 ± 0.008	A	8	5	4	3	7	2
*Dianthus deltoides* L.	Dde	Caryophyllaceae	P	1.1–1.6 × 0.7–1.0	0.106 ± 0.010	A	8	5	4	3	3	2
*Echium vulgare* L.	Evu	Boraginaceae	B/P	2.4–2.8 × 1.5–1.8	2.242 ± 0.048	A	9	6	3	4	8	4
*Gentiana cruciata* L.	Gcr	Gentianaceae	P	1.1–1.3 × 0.5–0.6	0.090 ± 0.003	B	7	6	4	3	8	3
*Hieracium pilosella* L.	Hpi	Asteraceae	P	2.1–2.3 × 0.5–0.5	0.147 ± 0.002	A	7	0	3	4	0	2
*Hypericum perforatum* L.	Hpe	Hypericaceae	P	1.0–1.2 × 0.5–0.6	0.076 ± 0.002	A	7	6	5	4	6	3
*Hypochaeris radicata* L.	Hra	Asteraceae	P	5.0–10.0 × 0.5–0.6	0.962 ± 0.025	A	8	5	3	5	4	3
*Plantago media* L.	Pme	Plantaginaceae	P	1.7–2.1 × 1.0–1.2	0.249 ± 0.007	B	7	0	7	4	7	3
*Potentilla recta* L.	Pre	Rosaceae	P	1.3–1.7 × 1.9–2.1	0.385 ± 0.018	B	9	7	5	3	5	2
*Prunella grandiflora* (L.) Scholler	Pgr	Lamiaceae	P	1.7–1.9 × 1.5-1.7	1.217 ± 0.031	B	7	0	5	3	8	3
*Rumex acetosella* L.	Rac	Polygonaceae	P	1.4–1.8 × 1.1–1.3	0.200 ± 0.013	A	8	5	3	4	2	2
*Stachys germanica* L.	Sge	Lamiaceae	B	1.9–2.2 × 1.4–1.6	1.392 ± 0.065	B	7	7	4	3	8	5
*Thymus serpyllum* L.	Tse	Lamiaceae	S	0.6–0.8 × 0.5–0.7	0.143 ± 0.004	A	7	6	5	2	5	1
*Verbascum thapsus* L.	Vth	Scrophulariaceae	B	0.8–1.0 × 0.5–0.6	0.131 ± 0.004	A	8	0	3	4	7	7
*Veronica teucrium* L.	Vte	Plantaginaceae	P	1.3–1.6 × 1.1–1.2	0.320 ± 0.022	B	7	6	5	3	8	2

**Notes.**

Nomenclature of studied species follows the Plant List (http://www.theplantlist.org).

a growth form of a given species, where: B –biennial herb, P –perennial herb, S –subshrub/semishrub.

b seed size follows the literature (Bojñnanský & Fargašová, 2007).

c seed weight was measured in this study (determined by weighing 100 air-dried seeds; mean ± SD, *n* = 4).

d soil preference of the studied species estimated prior the experimental phase (basing on criteria presented in the article), where: A—acidophilous species from dry acidic grasslands, B –basophilic species from xerothermic alkaline grasslands.

e ordinal scale (1–9) of Ellenberg’s Indicator Values (Ellenberg, 1991), where: L—light requirements ranging from 7 to 9, where 7 indicates well-lit/slightly-shaded conditions (c.a. 30% of relative illumination) and 9 indicates full light conditions (>50% of relative illumination); T—temperature requirements ranging from 5 to 7, where 5 where 5 indicates species preferring moderately cool to warm conditions (characteristic of montane and submontane conditions, mostly southern Fennoscandia) and 7 indicates species preferring warm conditions (characteristic of North European Plain); K—continentiality requirements ranging 2 to 5, where 2 indicates atlantic conditions and 5 indicates subatlantic to subcontinental conditions; F—soil moisture requirements ranging from 2 to 5, where 2 indicates dry soils and 5 indicates moist soils; R—soil pH requirements ranging from 2 to 9, where 2 indicates extremely acidic to acidic soils and 9 indicates extremely alkaline soils originating from limestones; N—soil nutrient requirements ranging from 1 to 7, where 1 indicates extremely infertile soils and 7 indicates fertile soils; 0—indifferent behaviour, wide amplitude or unequal behaviour in different areas; *-uncertain and not fully described behaviour.

Although functional ecology of seeds, including effects of habitat sieves (*e.g.*, edaphic factors) has recently gained more attention in plant ecology (Poschlod, 2020), there is only fragmental information about germination patterns that can be observed in temperate grasslands. For example, germination-related requirements pertaining to pH of calcicole and calcifuge plant species from temperate grasslands are hardly known. Furthermore, although influence of metals on the completion of germination was solicitously studied and reviewed, information pertaining to the role of Al as well as Fe and Mn (both in ionic and complexed forms) in this process is very scarce (Kranner & Colville, 2011). So far, some studies addressed only the problem of influence of Al toxicity on the ability of seeds to complete germination in some wild-living plant species (Abedi, Bartelheimer & Poschlod, 2013). Therefore, we performed the experiments to shed light on less studied aspects of germination ecology of the selected plant species from contrasting European grasslands (Table 1). The following hypotheses were addressed: (1) calcicole plant species (in terms of their centers of abundance) prefer alkaline pH while calcifuge plant species prefer acidic pH for completion of germination; (2) availability of iron (Fe-HBED and FeCl_3_) and manganese (Mn-HBED and MnCl_2_) impacts germination-related characteristics, and (3) increasing concentration of AlCl_3_ affects germination, whereby acidophilous species are more resistant than basophilous ones. The following questions were addressed: (1) do pH and availability of Fe, Mn and Al influence the ability of seeds to complete germination? (2) do HBED-chelated Fe and Mn affect germination to a greater extent than their chlorides? (3) is Al a real threat to germination-based revegetation?

## Materials & Methods

### Criteria of species selection and collection of seeds

The plant species used in this study were selected based on their habitat preferences (Table 1). In total, 20 species were selected (10 from acidic dry grasslands and 10 from alkaline xerothermic grasslands). Their occurrence on the respective sites in central Europe was also taken into consideration (Czyzewska, 1992; Mucina & Kolbek, 1993; Matuszkiewicz, 2001; Kącki, Czarniecka & Swacha, 2013; Hegi, 1931). According to the available literature (Ellenberg, 1991), all the selected species are light-loving plants that can be found in warm and relatively dry sites characterized with low nitrogen soil status (Table 1). Furthermore, the selected plant species belong to different plant families. With such a wide selection of species, we intended to examine the most common, as well as the most valuable, plants that can be found in the studied communities.

The propagules (fruits—achenes for the studied species from Asteraceae and Rosaceae, nutlets for the studied species from Boraginaceae and Lamiaceae –and seeds for rest of the studied species; therein after referred to as seeds) were manually collected from plants cultivated in the Didactic-Experimental Garden of Faculty of Biology and Environmental Protection, University of Lodz (51°78′N, 19°48′E; 223 m a.s.l.), at full maturity stage in summer, 2019 (exact timing depended on the species). The seeds were gathered from at least 20 healthy and representative plant individuals growing in homogenous and optimal conditions (matching individual requirements for growth and reproduction of each species). The seeds were stored in paper bags in the laboratory for 14 days (at a relative humidity of 30%). Then they were inspected (all malformed, discolored and damaged seeds were removed) and weighed (four lots of 100 randomly selected seeds). Dimensions of the seeds were taken from the literature (Bojñnanský & Fargašová, 2007). Then, the seeds were stratified in a refrigerator (5 °C) for 16 weeks.

### General germination procedures

The ability of seeds to complete germination was tested on glass Petri dishes (*ϕ* = 5 cm). The dishes were lined with two pieces of filter paper (Whatman no. 1; GE Healthcare, Chicago, IL, United States). The seeds were mixed before the tests in order to fulfill the randomization requirement. On each dish, 25 seeds were placed and moistened with 1.5 cm^3^ of the tested solutions. Subsequently, the dishes were sealed with parafilm to maintain homogenous moisture and humidity and placed in a germination cabinet. All experiments were conducted under identical conditions of fully-controlled thermo-photoperiod (16 h of light phase at 25 °C and 8 h of dark phase at 15 °C; light was supplied with white fluorescent lamps, PAR = 40 µmol m^−2^ s^−1^, measured with FluorPen; PSI, Drasov, Czech Republic). Those photo-thermal conditions were selected due to the fact that many of the species we studied complete germination in spring (Czarnecka, 2004), when air temperature is relatively high in central Europe. This thermoperiod was previously used in other multispecies investigations focusing on a similar topic (Tudela-Isanta et al., 2018) and it is within optimal temperature range allowing completion of germination of many species from the temperate zone, including those from temperate grasslands (Baskin & Baskin, 1988; Dürr et al., 2015; Ladouceur et al., 2019). Furthermore, based on the data pertaining to the seed weight of the species we studied (in most cases the seeds weighed <1.5 mg; Table 1), they are likely to be photoblastic positive or at least photoblastic neutral (Jankowska-Blaszczuk & Daws, 2007), which was confirmed by preliminary tests showing that all the species we studied were able to complete germination under the described conditions. All tested solutions were prepared using distilled water (conductivity <0.08 µS cm^−1^). The pH values of each solution (Table S1) were measured with digital pH-meter (CP-401; Elmetron, Zabrze, Poland). Completion of germination was counted daily for 21 d. During the experiments, the seeds were monitored for any signs of their unviability (seed softness and brownish embryo color), however no such situation was observed.

### Experiment 1: Effects of pH

Phosphate buffer (KH_2_PO_4_:Na_2_HPO_4_; 0.066 mol dm^−3^) was used in this study as a medium ensuring the desired pH. It was the buffer of choice due to its relatively wide range of available pH values. Moreover, it is not toxic to cells and its acidity does not change substantially with change of temperature. Furthermore, each used phosphate species co-occurs in soil with a given soil pH, *i.e.*, H_2_PO_4_^−^ occurs predominantly in acidic soils, while HPO_4_^2−^ in alkaline ones (Strawn, Bohn & O’Connor, 2020), thus it reflects the available forms of P in the studied grasslands (Leuchner & Ellenberg, 2017). The effect of pH on germination was studied within the pH range of 5.0–8.0 in increments of 1.0 pH unit (similar to a setup used elsewhere; Tudela-Isanta et al., 2018). The buffers were prepared using analytical grade salts. The experiment was run according to the setup described in ‘General Germination Procedures’.

### Experiment 2: Effects of ionic and complexed iron

Fe-HBED chelate (*N*,*N*’-di(2-hydroxybenzyl)ethylenediamine-*N*,*N*’-diacetic acid iron(III) sodium salt; 7% Fe; synthesized according to European Patent Application EP20461587.6 by PPC ADOB, Poland, and authenticated using LC-MS/MS, FTIR and UV-VIS analyses; Fig. S1) was used as a complexed form of Fe (chelation rate 1:1 with favored Fe(III) oxidation state). This agent is characterized by high stability under a wide range of conditions, including pH, thus it can be used as a tool simulating natural metal chelation under high soil pH. Anhydrous FeCl_3_ of analytical quality was used as the ionic form of Fe^3+^. The effect of Fe was evaluated using isomolar concentrations of both compounds (0, 5 and 25 µmol dm^−3^). This range of concentrations was the same as that used in our previous studies evaluating physiological response of plants at vegetative stage (Wala et al., 2020). These concentrations were also within the range maintaining optimal plant growth under hydroponic conditions (Venturas et al., 2014; Kasozi et al., 2019) and are higher than the concentration of available Fe in a soil solution of Rendzinas (picomolar/nanomolar values; Bothe, 2015). The experiment was run according to the setup described in ‘General Germination Procedures’.

### Experiment 3: Effects of ionic and complexed manganese

The complexed form of Mn (chelation rate 1:1 with favored Mn(III) but possible Mn(II) oxidation state; Pinto et al., 2019) was supplied as Mn-HBED (*N*,*N*’-di(2-hydroxybenzyl)ethylenediamine-*N*,*N*’-diacetic acid manganese (II) sodium salt; 7% Mn; synthesized by PPC ADOB, Poland). HBED was selected as Mn-complexing agent in order to reduce bias resulting from comparison of different chelators (in this case Fe-HBED vs Mn-HBED). Furthermore, high stability of HBED under alkaline conditions simulates chelation process in the studied type of community (alkaline xerothermic grassland).

Mn-HBED was synthesized as following: a 250 cm^3^ flask with a magnetic stirrer, a pH-meter and a reflux condenser was charged with 100 g of water and 23.96 g (0.05 mol) of *N,N’*-di(2-hydroxybenzyl)ethylenediamine-*N,N’*-diacetic acid (HBED) monohydrochloride (purity 88.6%). Next, 1 mol dm^−3^ solution of sodium hydroxide (NaOH) was added to the slurry until pH 7 was reached. After complete homogenization, 10.39 g (0.0525 mol) of MnCl_2_ ⋅ 4H_2_O (purity 99%) in 30 ml of water was added in portions. During the addition of MnCl_2_ ⋅ 4H_2_O, pH was maintained in range of 6–8 using 1 mol dm^−3^ solution of NaOH. After the complete addition of MnCl_2_ ⋅ 4H_2_O, mixture was stirred for 1 h at room temperature. Then, the mixture was alkalized to pH 11 using a 1 mol dm^−3^ solution of NaOH. The solution was left without stirring for 24 h in darkness to allow excess Mn to precipitate as oxide then filtered through a cellulose filter and the solvent was removed *in vacuo*. Finally, 23.50 g of *N,N’*-di(2-hydroxybenzyl)ethylenediamine-*N,N’*-diacetic acid, manganese(II) sodium salt, were obtained. The product assay was 7.33% of manganese according to EN 16963:2018-03. Mn-HBED was authenticated using LC-MS/MS, FTIR and UV–VIS analyses (Fig. S1).

The ionic form of Mn was supplied with analytical grade MnCl_2_ ⋅ 4H_2_O. The effect of Mn was evaluated using concentrations equal to those used in Experiment 2 (0, 5 and 25 µmol dm^−3^). These concentrations were used in order to (1) compare the effects of Fe and Mn and (2) simulate Mn-over-supplemented environment, more Mn-rich than a soil solution (where average availability of Mn ranges c.a. 0.1–10 µmol dm^−3^ soil solution and in near-alkaline and acidic soils is close to 0.4 and 16.0 µmol dm^−3^ soil solution, respectively; Marschner, 1988; Shao et al., 2017) and artificial media optimal for soil-less plant cultivation (*e.g.*, Hoagland solution, where Mn concentration in full strength medium is equal to 9 µmol dm^−3^; Asher & Edwards, 1983). The experiment was run according to the setup described in ‘General Germination Procedures’.

### Experiment 4: Effects of aluminum

The test was conducted according to the available data reporting a nonlinear relation between exchangeable Al and the pH of soil (Abedi, Bartelheimer & Poschlod, 2013). Analytical grade AlCl_3_ ⋅ 6H_2_O was used as source of ionic Al (Abedi, Bartelheimer & Poschlod, 2013). The effect of Al on seed germination was evaluated using 0.00, 0.01, 0.10, 1.00 and 10.00 mmol dm^−3^ solutions, which reflects the availability gradient of this element from alkaline to acidic soils (Abedi, Bartelheimer & Poschlod, 2013). Tested solutions were prepared using the serial dilution method. The experiment was run according to the setup described in ‘General Germination Procedures’.

### Calculation of germination-related parameters

To determine reactions of the species we studied, the final germination percentage (FGP; ranging 0–100%) and index of germination velocity (IGV, known as modified Timson’s index, ranging 0–100; Khan & Ungar, 1997) were calculated. The greater values of FGP and IGV, the greater ability to complete germination or the more rapid germination, respectively. The IGV was calculated with the germinationmetrics package v. 0.1.3 (Aravind et al., 2020) run in R software (v. 3.5.2, 64 bit version; R Core Team, 2018) using preprogrammed equations of the package.

### Statistical analysis

All experiments were replicated four times (*n* = 4). Normality of the data was analyzed with the Kolmogorov–Smirnov’s test and homogeneity of variances was analyzed with the Brown–Forsythe’s test. To detect differences among treatments within a given species, one-way ANOVA followed by the Bonferroni post-hoc test was mounted (differences were accepted as statistically significant at *p* < 0.05). To inspect effects of the studied factors, two-way ANOVA (Experiment 2 and Experiment 3) and three-way ANOVA (comparison between Experiment 2 and Experiment 3) were used. Differences between effects of isomolar solutions of Fe and Mn (Experiment 2 and Experiment 3, respectively) were detected with two-way ANOVA followed by the Bonferroni post-hoc test (differences were accepted as statistically significant at *p* < 0.05). Correlation between seed weight and germination reaction was calculated with Spearman’s sum rank test. Analysis was conducted for all tested species (*n* = 20) as well as for separated groups of acidophilous (*n* = 10) and basophilous (*n* = 10) species. For each FGP value, the data were normalized to FGP values recorded at pH = 7 (Experiment 1) or control (null concentration) conditions (Experiments 2–4). Correlation was considered as statistically significant at *p* < 0.05. All statistical analyses were performed with Statistica™ v. 13.3 (Tibco Software Inc.; Palo Alto, CA, USA).

To find out if there are similarities between the studied species (segregation of species on the basis of germination strategies), FGP-based hierarchical cluster analysis (HCA) was performed, merging data from Experiments 1–4. For each species, in order to reduce bias from the species-specific reaction (namely due to differences in completion of germination under control conditions), the data were normalized by calculation of ratio of FGP value at given treatment and FGP value recorded at pH = 7 (Experiment 1; due to neutral acidity and the most proportional KH_2_PO_4_: Na_2_HPO_4_ ratio) or control (null concentration) conditions (Experiments 2–4) as it was done for calculation of correlations. Phenograms were constructed using Ward’s method of row clustering and Manhattan distance and the tightest clusters were presented first. HCA analysis was performed using ClustVis online tool (Metsalu & Vilo, 2015) with mean values from normalization, as proposed recently (Chen et al., 2020).

## Results

### Experiment 1: Effects of pH

**Figure 1:**
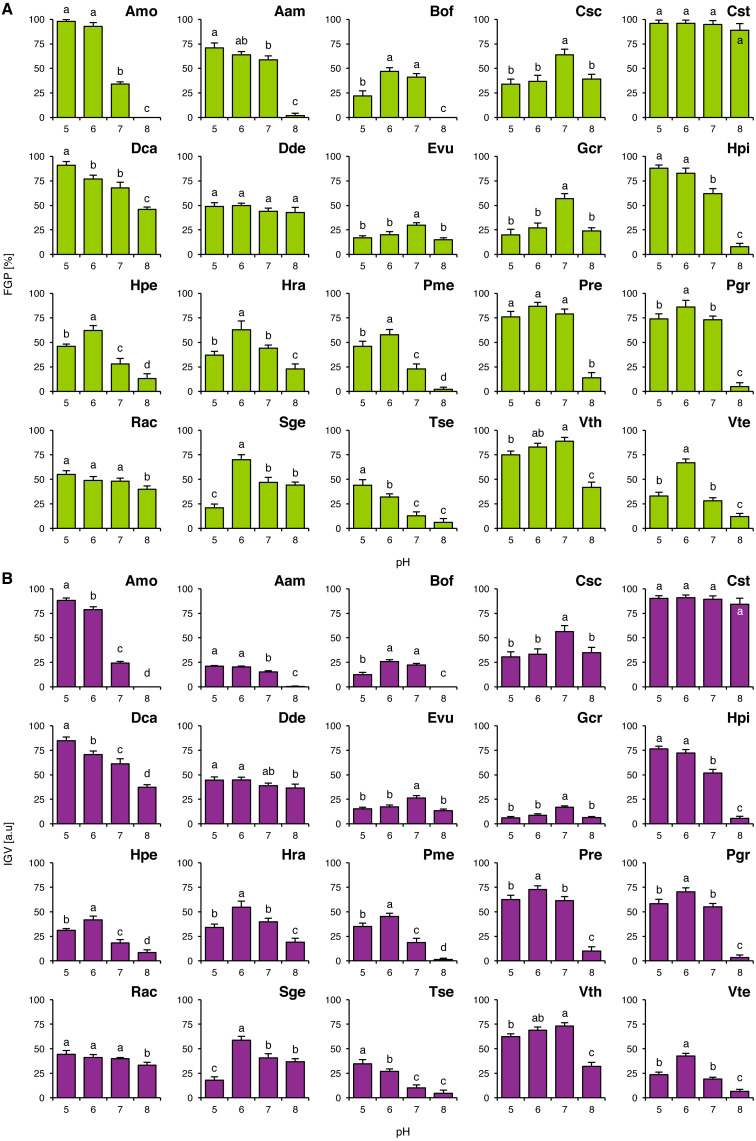
Effect of pH on the final germination percentage (FGP; A) and index of germination velocity (IGV; B) of the tested species from acidic dry and alkaline xerothermic grasslands. Values are the mean ± SD (*n* = 4). Different letters indicate significant differences between groups (ANOVA with Bonferroni post-hoc test, *p* < 0.05).

FGP values did not depend on pH of the buffered solutions in Cst and Dde (Fig. 1A), whilst in the other studied species they were differentially regulated. Acidophilous species showed preference to acidic conditions (Amo, Hpi, Hpe, Hra, Tse and Vth) and neutral conditions (Evu) or the preference was not clear (Rac; Fig. 1A). On the other hand, basophiles showed 4 general types of reactions: (1) preference for near-neutral pH (Csc and Gcr), (2) good completion of germination at acidic and neutral pH with strongly marked inhibition of completion of germination at alkaline pH (Aam, Bof, Pme, Pre, Pgr and Vte), (3) relatively wide tolerance of the tested pH values of the buffered solutions with marked acidophilism (Dca) and (4) strong preference to neutral and alkaline conditions with marked decrease in FGP under acidic conditions (Sge) (Fig. 1A). Interestingly, Tse showed marked decrease in FGP at optimal pH value, when compared to null concentrations in Experiments 2–4 (Fig. 1).

IGV values were nearly proportional to corresponding FGP values with two exceptions. In the case of Aam and Gcr completion of germination was slow (Fig. 1B). Germination speed in Gcr did not dependent on the buffer composition, as this species completed germination with similar velocity in Experiments 2–4, whilst in Aam this was probably caused by the compounds present in the buffered solutions (KH_2_PO_4_ and Na_2_HPO_4_) or their concentration (when compared to IGV values from Experiments 2–4).

### Experiment 2: Effects of ionic and complexed iron

**Figure 2:**
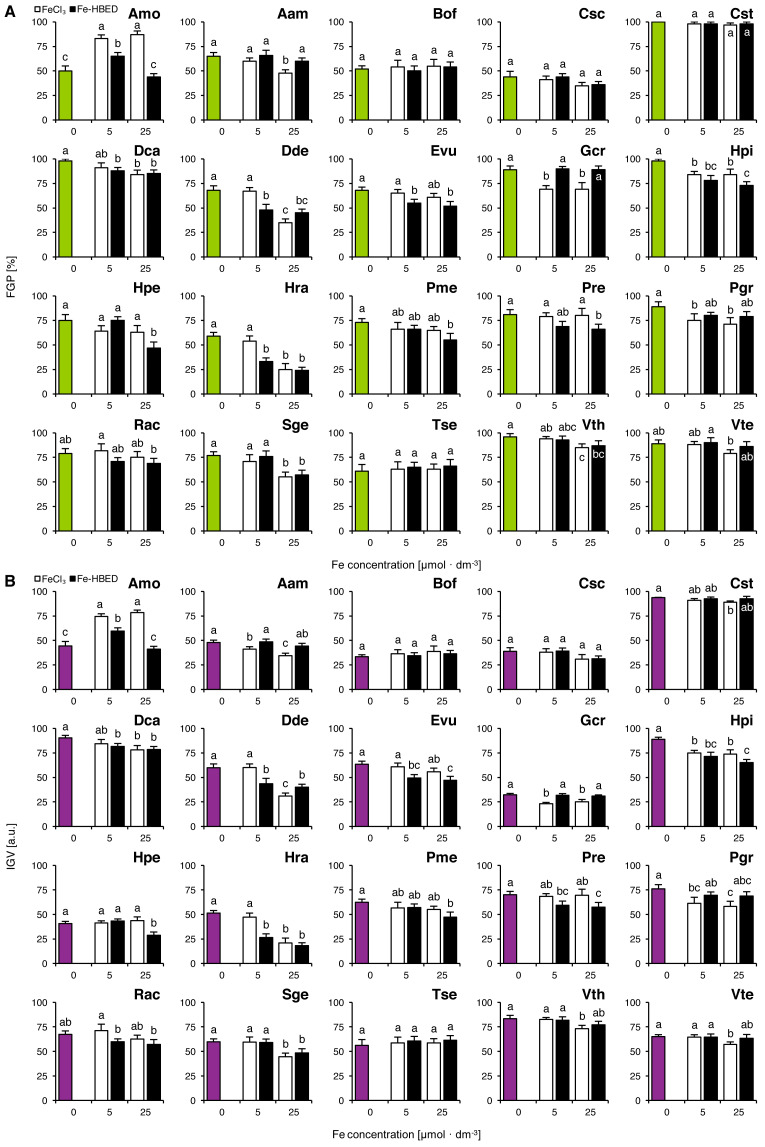
Effect of ionic and chelated iron (FeCl_3_ and Fe-HBED) on the final germination percentage (FGP; A) and the index of germination velocity (IGV; B) of the tested species from acidic dry and alkaline xerothermic grasslands. Values are the mean ± SD (*n* = 4). Different letters indicate significant differences between groups (ANOVA with Bonferroni post-hoc test, *p* < 0.05).

The tested Fe-containing compounds affected completion of germination of the studied species except that of Bof, Csc, Cst and Tse (Fig. 2A). Considering the FeCl_3_-treated seeds, reduction of completion of germination was observed both after the treatment with 5 µmol dm^−3^ (Gcr, Hpi and Pgr) and with 25 µmol dm^−3^ (Aam, Dca, Dde, Gcr, Hpi, Hra, Pgr, Sge, Vth and Vte; Fig. 2A). Similar results were observed for Fe-HBED-treated seeds, as the solution of 5 µmol dm^−3^ negatively affected completion of germination in Dca, Dde, Evu, Hpi and Hra, whilst that of 25 µmol dm^−3^ reduced completion of germination in Dca, Dde, Evu, Hpi, Hpe, Hra, Pme, Pre, Sge and Vth (Fig. 2A). Both FeCl_3_ (5 and 25 µmol dm^−3^) and Fe-HBED (5 µmol dm^−3^) positively affected completion of germination in Amo (Fig. 2A).

Effects of the tested conditions on FGP and IGV values were similar (Fig. 2B). With the exception of Gcr which germinated slowly but very successfully (Fig. 2A).

Two-way ANOVA showed that the type of Fe-bearing compound influenced FGP values in nine species (Amo, Aam, Evu, Gcr, Hpi, Hra, Pre, Pgr and Rac), whilst the dose of tested compound affected 10 species (Amo, Aam, Csc, Dca, Dde, Hpe, Hra, Sge, Vth and Vte; Table S2). Interaction between the type and the dose of the tested compounds affected significantly 4 species in total (Amo, Dde, Hpe and Hra; Table S2). IGV values were significantly affected by Fe type in 11 species (Amo, Aam, Cst, Evu, Gcr, Hpi, Hpe, Hra, Pre, Pgr and Rac) and by Fe dose in 12 species (Amo, Aam, Csc, Dca, Dde, Hpe, Hra, Pme, Rac, Sge, Vth and Vte; Table S2). Interaction between these factors affected IGV values in four species (Amo, Dde, Hpe and Hra; Table S2).

### Experiment 3: Effects of ionic and complexed manganese

**Figure 3:**
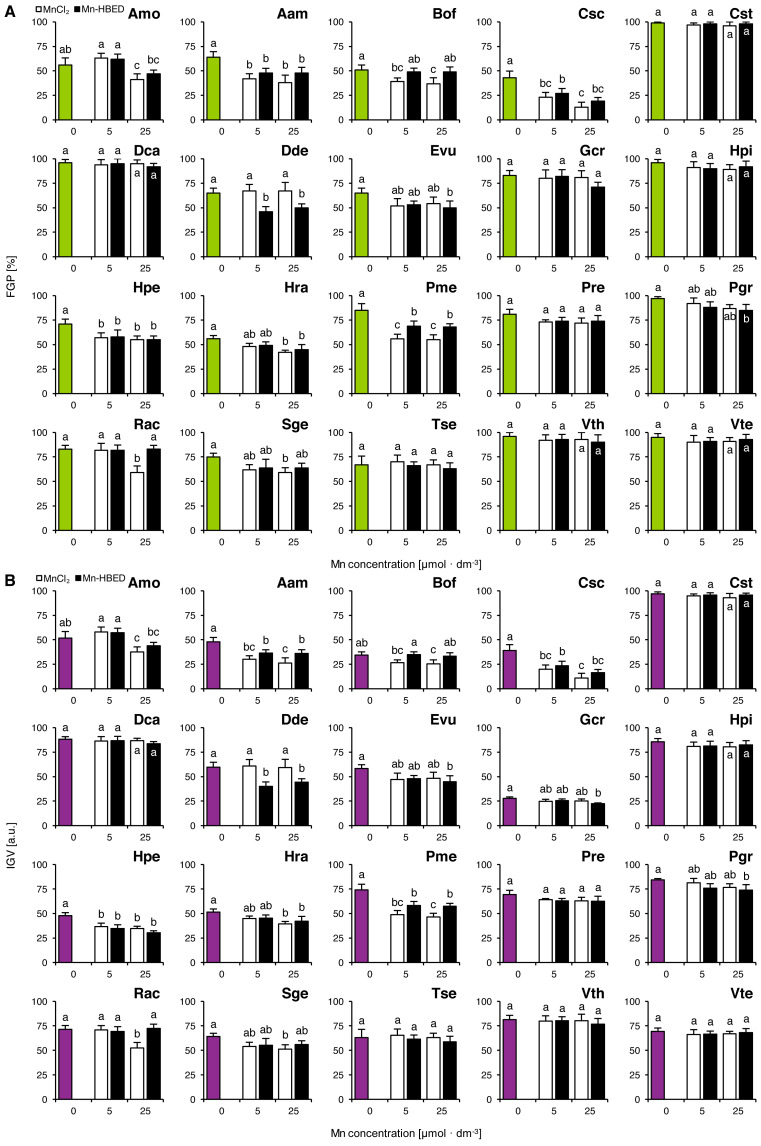
Effect of ionic and chelated manganese (MnCl_2_ and Mn-HBED) on the final germination percentage (FGP; A) and index of germination velocity (IGV; B) of the tested species from acidic dry and alkaline xerothermic grasslands. Values are the mean ± SD (*n* = 4). Different letters indicate significant differences between groups (ANOVA with Bonferroni post-hoc test, *p* < 0.05).

Mn treatments had no effects on FGP values in Cst, Dca, Gcr, Hpi, Pre, Tse, Vth and Vte (Fig. 3A). On the other hand, MnCl_2_ solution of 5 µmol dm^−3^ had a negative effect on the ability of seeds to complete germination in Aam, Bof, Csc, Hpe and Pme (Fig. 3A). Decreases in FGP values were observed in Amo, Aam, Bof, Csc, Hpe, Hra, Pme, Rac and Sge due to application of 25 µmol dm^−3^ MnCl_2_ solutions (Fig. 3A). Negative effects of Mn-HBED were also observed in the species treated with 5 µmol dm^−3^ (Aam, Csc, Dde, Hpe and Pme) as well as with 25 µmol dm^−3^ solutions (Aam, Csc, Dde, Evu, Hpe, Hra, Pme and Pgr; Fig. 3A).

The changes of IGV values were similar to those in FGP (Fig. 3B). Similar to what was shown in Experiment 2, Gcr proved to be an exceptionally slow germinator (Fig. 3B).

Two-way ANOVA showed that the type of Mn-containing agent affected five species (Aam, Bof, Dde, Pme and Rac) and Mn dose affected significantly four species (Amo, Csc, Hra and Rac; Table S2). Only one species was affected significantly by the interaction between the tested factors (Rac; Table S2). Similar effects were observed for IGV values (where Aam, Bof, Dde, Pme and Rac were affected by Mn type and Amo, Csc, Hra and Rac were affected by Mn dose; Table S2), but in the case of interaction between Mn type and dose, two species were affected significantly (Gcr and Rac; Table S2).

### Comparison of iron and manganese as stimuli for germination

Five species (Cst, Pre, Sge, Tse and Vth) had similar tolerance to both metals (Table S3). Seven species completed germination better in Fe than in Mn solutions (Amo, Aam, Bof, Csc and Rac in the case of chlorides; Aam, Csc, Gcr and Hpe of HBED-chelated forms) while for the others quite the opposite was the case (*e.g.*, Dca for 25 µmol dm^−3^ of chloride; Dde, Hra and Vte for 25 µmol dm ^−3^ of chlorides; Pgr for both doses of chlorides; Hpi and Rac for both concentrations of HBED-chelated forms; Table S3).

Three-way ANOVA showed that the element (Fe/Mn) affected FGP values in 12 species (Amo, Aam, Bof, Csc, Dca, Dde, Evu, Hpi, Hpe, Hra, Pgr and Vte), the type of metal bearing compounds influenced FGP values significantly in 11 species (Amo, Aam, Bof, Csc, Dde, Evu, Gcr, Hpi, Hra, Pme and Pre) and the dose affected FGP values in nine species (Amo, Aam, Csc, Dde, Hpe, Hra, Rac, Sge and Vth; Table S4). Interactions between two factors affected 5–11 species and interaction between all three factors affected only five species (Amo, Dde, Hpe, Hra and Rac; Table S4). Very similar effects were observed for IGV values (Table S4).

### Experiment 4: Effects of aluminum

**Figure 4:**
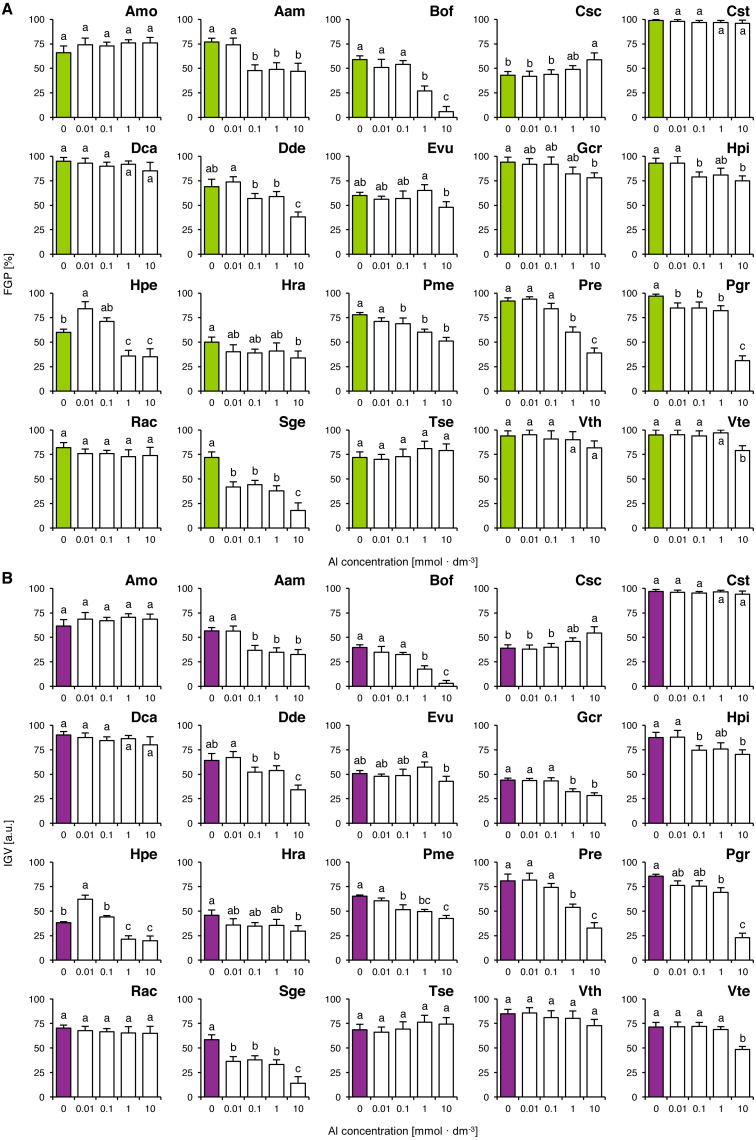
Effect of aluminum (AlCl_3_) on the final germination percentage (FGP; A) and index of germination velocity (IGV; B) of the tested species from acidic dry and alkaline xerothermic grasslands. Values are the mean ± SD (*n* = 4). Different letters indicate significant differences between groups (ANOVA with Bonferroni post-hoc test, *p* < 0.05).

Six species (Amo, Cst, Dca, Rac, Tse and Vth) completed germination well in all tested concentrations of Al, as their FGP values did not differ significantly (Fig. 4A). The solutions ≥ 0.01 mmol dm^−3^ inhibited completion of germination in Pgr and Sge, ≥ 0.1 mmol dm^−3^ and greater solutions inhibited completion of germination in Aam, Dde, Hpi and Pme, ≥ one mmol dm^−3^ and greater solutions reduced FGP values in Bof, Hpe and Pre and 10 mmol dm^−3^ solutions reduced FGP values in Gcr, Hra and Vte (Fig. 4A). Interestingly, Al-stimulated completion of germination was observed in Hpe (0.01 mmol dm^−3^) and in Csc (10 mmol dm^−3^; Fig. 4A). The observed patterns of FGP responses were similar to those of IGV (Fig. 4B).

### Classification of germination requirements and relations of seed traits to germination

Seed weight was negatively correlated with FGP values only when the seeds were subjected to 5 µmol dm^−3^ MnCl_2_ (R = −0.447; *p* = 0.048; Table S5). IGV values were also negatively correlated with weight only when the seeds were subjected to 0.01 mmol dm^−3^ AlCl_3_ (R = −0.503; *p* = 0.024 for all the studied species and R = −0.648; *p* = 0.043 for acidophiles; Table S5). All other correlations were insignificant (*p* > 0.05; Table S5).

**Figure 5:**
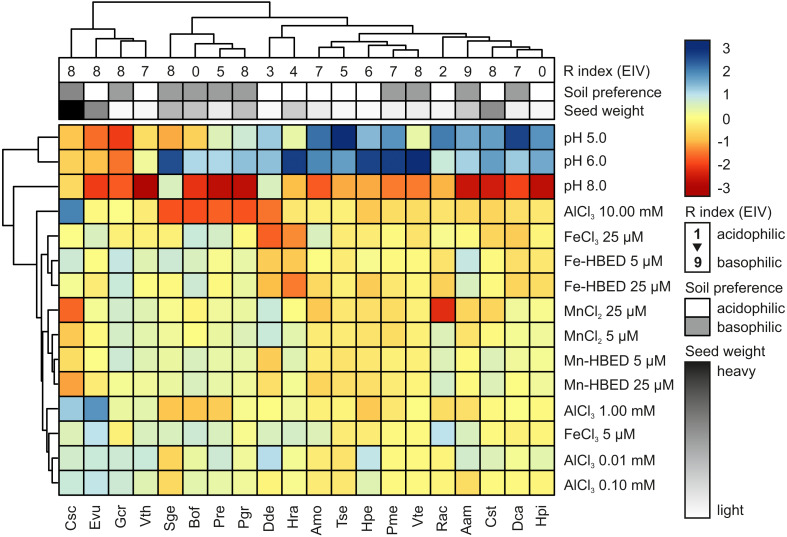
Hierarchical clustering analysis (HCA) of germination reactions (FGP) of the studied species to the tested conditions. Analysis was conducted using data from Experiments 1–4. For each species, the data were normalized to FGP values recorded at pH = 7 (Experiment 1) or control (null concentration) conditions (Experiments 2–4). Three levels of annotations represents data presented in Table 1 (R Ellenberg’s Indicator Value, estimated soil preference and seed weight). Blue and red colors in scale bar represents stimulation and inhibition of FGP, respectively. Phenograms were constructed using Ward’s method of row clustering and Manhattan distance.

Hierarchical cluster analysis (HCA) showed that acidity and extremely high concentration of Al (10.00 mmol dm^−3^) were major factors allowing clustering of the studied species, whilst the other studied treatments had only minor contribution to species segregation (Fig. 5). According to the HCA, five major groups of species were distinguished: (1) species with preference for sub-neutral acidity (pH = 6) that are able to tolerate high doses of Al (Csc, Evu, Gcr and Vth); (2) species with marked inhibition of germination completion at pH = 8 and reduced ability to complete germination in the presence of Al (Sge, Bof, Pre and Pgr); (3) species with rather wide tolerance to acidity and not strongly affected by Al (Dde and Hra); (4) species with marked preference for acidic conditions and no obvious reaction to Al (Amo, Tse, Hpe, Pme and Vte); and (5) species that prefer acidic conditions (with marked susceptibility to alkalization) and no reaction to Al (Rac, Aam, Cst, Dca and Hpi; Fig. 5). The estimated soil preference and seed weight were not good predictors of completion of germination under the tested conditions, as clustering did not distinguished sharp and homogenous groups (Fig. 5). On the other hand, three first clusters were relatively well segregated considering R Ellenberg’s Indicator Value (R EIVs), but the remaining two clusters did not show any logical pattern (Fig. 5).

## Discussion

It is commonly accepted that grasslands established on alkaline calcareous soils are composed of calcicole/calciphile/basophile plant species, while those established on acidic sandy soils are composed of calcifuges/calciphobes/acidophiles (Bothe, 2015). However, this concept was only occasionally tested to show its viability (Bothe, 2015), especially considering germination requirements. Although drastic changes of soil chemistry (*e.g.*, pH –(Wagner et al., 2017) –and availability of nutrients, most remarkably Fe, Mn and Al) in Rendzinas and Podzols are not very likely, slightly varying physical-chemical properties of soil (including its patchy structure; Cain et al., 1999) create a mosaic of microhabitats which contributes to plant diversity (Leuchner & Ellenberg, 2017). It suggests that some species within a given habitat type can be more/less calciphilic (or acidophilic) than others or show an undifferentiated reaction to soil pH and availability of the studied metals (in terms of germination requirements). The presented work is in line with this conception. It must be however noted that further validation of the presented findings on annual dicotyledonous plants as well as grasses is needed in order to fully describe the studied plant communities.

The recorded data clearly demonstrated that ability of the studied species to complete germination was pH-dependent (hypothesis 1). Even the species considered as undeniable acidophiles and basophiles (considering their centers of abundance) are able to complete germination under the conditions which are not their typical ones (*e.g.*, Aam, Bof, Csc, Cst, Dca, Dde, Evu, Pre, Pgr, Rac, Vth and Vte). Furthermore, for some species that can be found both in xerothermic and dry grasslands (Cst, Dca, Hpe, and Evu) the amplitude of soil pH allowing them completion of germination is relatively wide. However, it should be interpreted with caution because mismatch between the conditions allowing completion of germination and those permitting optimal vegetative growth are known, which was shown previously, *e.g.*, for soil moisture (Wilman et al., 2014). Overall, it is worth noting that both alkaline xerothermic and acidic dry grasslands are composed of (1) species with pH requirements for completion of germination exactly matching the pH of the substratum on which they can be found, (2) pH-undifferentiated species (wide theoretical regeneration niche) and (3) species characterized with marked pH preferences however having also partial ability to complete germination under non-optimal conditions. It is known that there is diversity of germination strategies within the flora of alkaline and acidic grasslands, including such adaptations as reaction to nitrogen (Wala, Kołodziejek & Patykowski, 2021), ability to withstand thermal aging (Tausch et al., 2019) and complete germination under a given thermoperiod (Tudela-Isanta et al., 2017). Although previous studies suggested that alpine calcicoles and calcifuges possessed group-specific adaptations allowing completion of germination under acidity matching their centers of abundance (Tudela-Isanta et al., 2018), a homogeneous reaction within a group is probably not as plausible in lowland temperate grasslands (Margreiter et al., 2020). Our study is in agreement with the conception of heterogeneity of germination strategies within calcicole/calcifuge groups, as it clearly shows that a potential germination-based regeneration niche of several species (*e.g.*, Aam, Cst, Dde, Pgr, Rac) is wide and does not match their realized niches and centers of abundance. These species cannot be usually found under non-typical soil conditions, which probably results from weak competitiveness at the vegetative stage. For example, Rac is known to be a pH-indifferent germinator (Yazdi et al., 2013), but is not able to win competition with basophilic species during establishment on calcareous soils, also due to susceptibility to Fe-dependent limitations (Hutchinson, 1967; Leuchner & Ellenberg, 2017). On the other hand, some calcicole species (occurring naturally on alkaline soils, *e.g.*, Rendzinas) showed exceptional tolerance to low pH at the germination stage (*e.g.*, Aam and Pgr) but they were not able to develop correctly under high soil acidity of Podzols (Wala et al., 2020).

It must be noted that the composition of buffer solution used for studies on pH optimum for completion of germination can affect the results (Ma et al., 2015). Due to the fact that we used in this study isomolar buffers containing only phosphates, the results can be the effect of any of the three traits of the used buffers, namely differing acidity (pH) or phosphate ratio (HPO_4_^2−^: H_2_PO_4_^−^) or ratio of Na^+^: K^+^. Thus, species limited by the highest pH values used in this study (calcicole Aam, Amo, Bof, Pre, Pgr and Pme and calcifuge Hpi) may also additionally encounter adverse effects of HPO_4_^2−^ and/or Na^+^. As both phosphate forms can be utilized by plants; Schachtman, Reid & Ayling, 1998), Na-caused toxicity is more probable explanation. This element (whose availability can be increased due to agronomical nutrient loadings; Núez-Delgado, López-Periago & Díaz-Fierros Viqueira, 2002) can disturb the germination-based revegetation of the studied types of grasslands because it is relocated with percolate during soil surface runoff across slopes (Schulze et al., 2019) from arable fields to lower areas. Sodic-saline conditions were repeatedly shown to deteriorate edaphic conditions, ability of seeds to complete germination, plant performance (Läuchli & Grattan, 2012) and regeneration sites (Ma et al., 2015). Therefore, it can be treated as another threat for the species we studied. Additionally, it is worth noting that alkaline soils, on which xerothermic grasslands are settled in Central Europe, are rather non-sodic (Tóth, Montanarella & Rusco, 2008). It partially explains why the studied xerothermic calcicoles are probably not adapted to elevated Na concentrations and can suffer from adverse effects of this element. It provokes further studies concerning the effects of sodic-alkaline environment on the ability of seeds to complete germination.

Influence of Fe on the ability of seeds to complete germination was only occasionally studied (El Rasafi et al., 2016; Reis et al., 2018). For example, 20 µmol dm^−3^ solution of FeCl_3_ was shown to have no significant effect on the ability to complete germination of crop plants such as *Triticum aestivum* L. and *Phaseolus vulgaris* L. (El Rasafi et al., 2016) and similar reactions were observed for Bof, Csc, Cst and Tse investigated in this study. On the other hand, a reduction of FGP due to application of ionic Fe (given as FeSO_4_) was observed in *T. aestivum* treated with solutions of c.a. 90–143 µmol Fe^2+^ ⋅ dm^−3^ (Reis et al., 2018). Interestingly, there are also some available data suggesting that completion of germination in rice (*Oryza staviva* L.) could be accelerated due to application of FeSO_4_ (c.a. 6.6 mmol Fe^2+^ ⋅ dm^−3^; Wang et al., 2020). In the presented study, detrimental changes pertaining to germination parameters due to application of FeCl_3_ could be the result of Fe itself or Cl^−^ load (15/75 µmol Cl^−^ ⋅ dm^−3^). However, it is worth noting that due to low concentrations of the tested solutions, effects of osmotic pressure are not high enough to affect germination at imbibition phase, which can be extrapolated from numerous studies showing effects of polyethylene glycol-simulated osmoticum on the ability of seeds to complete germination (Chamorro & Moreno, 2019; Yi et al., 2019). This strongly suggests that several studied species (Aam, Dca, Dde, Gcr, Hpi, Hra, Pgr, Vth and Vte) are susceptible to overdose of Fe^3+^, as considerable concentrations of Cl^−^ (up to 30 mmol dm^−3^) are known to have no or marginal influence on the germination process (Abedi, Bartelheimer & Poschlod, 2013). Although mechanism of Fe toxicity still needs detailed studies, it can be hypothesized that reduced completion of germination due to supraoptimal doses of Fe can be the result of Fe-dependent disturbances in a cell cycle (Reis et al., 2018) resulting from oxidative stress caused by photo-Fenton and Fenton-like reactions catalyzed by Fe (Rincón & Pulgarin, 2006; Ahile et al., 2021) and/or non-oxidative ion-specific toxicity. In nature, release of Fe species from Fe-bearing minerals is to a great extent controlled by soil pH (Strawn, Bohn & O’Connor, 2020). In fact, concentration of Fe^3+^ released from Fe oxides under alkaline conditions (pH = 8.5) is very low and becomes significant only at remarkably acidic conditions (Robin et al., 2008). It partially explains why some undeniable calcicoles (Aam, Dca, Gcr, Pgr, Sge, and Vte) are negatively influenced by ionic Fe. However, some studied calcifuges (Cst, Dde, Hpi, Hra and Vth) also seem to be prone to Fe-dependent limitations at the germination stage (hypothesis 2). It can be proposed that Fe soil status is an additional but minor ecological sieve filtering establishment of new individuals in the studied communities. However, the way in which Fe becomes a potential limiting factor in each of the studied types of communities may vary. For example, in the case of dry acidic grasslands, availability of iron can be shaped by succession (Sparrius, Sevink & Kooijman, 2012). Another way to increase availability of Fe on soil surface is severe soil disturbance (caused by anthropogenic and natural activities) and eventually displacement of Fe-rich hardpan, which is then solubilized. A different mode of action is more plausible in alkaline xerothermic grasslands, where Fe chelation due to biological activity at the local scale is probably the major factor influencing its availability (Singer, Schwertmann & Friedl, 1998; Gries & Runge, 1992; Ferreira et al., 2019). Although not much is known about the quantitative siderophoral status of Rendzinas, studies on Podzols showed that this kind of soil contains nanomolar concentrations of a given siderophore/chelator (a dozen or so nanomoles on average; Winkelmann, 2007; Ahmed & Holmström, 2015). Thus, it is very likely that the total pool of Fe chelators (and thus chelated Fe as there is a linkage between concentration of chelators and chelated Fe; Liu & Millero, 1999) is at the micromolar level in both soils (Robin et al., 2008), but their biological recovery from the alkaline soil may be even harder than from the acidic one in some cases due to lime-dependent sequestration (Boiteau et al., 2020). In the context of the presented study, such chelator-based Fe dissolution in soil may have some impact on germination-based revegetation, affecting the studied plants in a species-specific manner.

The role of ionic and complexed forms of Mn on geochemical processes and life (including plant ontogenesis, *e.g.*, a germination process) still needs elucidation (Duckworth, Bargar & Sposito, 2009). To date, it has been demonstrated that Mn (solutions of c.a. 5–91 µmol Mn^2+^ ⋅ dm^−3^ given as MnSO_4_) did not significantly affect FGP of lettuce (*Lactuca sativa* L.) seeds sown on Hoagland medium (Liu, Zhang & Lal, 2016). On the other hand, the completion of germination of *Nicotiana tabacum* L. seeds was significantly reduced up to 75% after application of 2–20 mmol Mn^2+^ ⋅ dm^−3^ (given as MnSO_4_) on Murashige and Skoog medium (Santandrea, Schiff & Bennici, 1997). Reduction of FGP due to increasing availability of Mn seems to be species-specific, as plants can differ in their reaction to Mn concentration at the germination stage (*e.g.*, *Arabidopis thaliana* L. and *Arabis paniculata* Franch. subjected to 1 and 10 mmol Mn^2+^ ⋅ dm^−3^ given as MnCl_2_; Tang, Tao & Li, 2021). On the basis of the presented study it can be stated that Mn species-specifically influenced the ability of seeds to complete germination (hypothesis 2). As eight studied species did not suffer from the negative effects of Mn (Csc, Dca, Gcr, Hpi, Pre, Tse, Vth and Vte) and there was no sharply pronounced pattern of reaction to the tested stimuli within each ecological group, it can be proposed that the reaction to Mn availability is individual for each species, which further implies that microhabitats (in terms of Mn availability gradient or hotspots) can play a role in establishment of the studied plants. It is however worth noting that oxidative state of Mn also should not be underestimated, as chemical structure HBED is believed to favor Mn(III) state (similarly to other Mn chelators; Duckworth, Bargar & Sposito, 2009) due to two phenolato donors of this chelator (Pinto et al., 2019) and/or oxidation under oxygen environment (Wahsner et al., 2019), although occurrence of Mn(II) state is also possible. From an environmental point of view, it is believed that complexed forms of Mn prevail in soil solution at neutral pH (>90% of Mn pool), while under acidic conditions, free Mn^2+^ is probably the predominant form of this element (>70% of Mn pool; Ritchie, 1989). Slightly reduced FGP and IGV values in basophilic species (Aam, Bof, Csc, Pme, Pgr and Sge) treated with Mn-HBED as well as in acidophilic ones (Amo, Evu, Hpe, Hra and Rac) treated with MnCl_2_ suggest that the predominant form of Mn in each type of grasslands plays a role in assembly of plant community.

Interplay between endogenous Fe and Mn is known to influence the seed germination process (Eroglu et al., 2017). The present study showed that exogenous Fe and Mn also affected the ability of seeds to complete germination. Additionally, considering the possible application of our results, it seems that the chelated forms of the studied metals (Fe-HBED and Mn-HBED) are not more detrimental to the germination process in the studied plants when compared to their respective ionic forms (isomolar solutions). This indicates that reasonable usage of HBED-chelated Fe and Mn should not pose a risk to terrestrial vegetation, which was suggested for other Fe chelates (European Food Safety Authority, 2013).

Limitations caused by increased Al availability were widely shown to occur in many terrestrial plant species (Singh, 2017). Inhibition of root elongation caused by destabilization of cell homeostasis is the most recognized sign of Al toxicity at the early stages of plant growth (Alves Silva et al., 2014). However, relatively little is known about the direct influence of Al on the ability of seeds to complete germination. To date, the most detailed study on this topic has been conducted on plants from dry temperate grasslands that can be found along an acidity gradient (Abedi, Bartelheimer & Poschlod, 2013). Among the 15 investigated species, a little more than half of them were resistant to Al toxicity at the germination stage (which was also shown for Amo, Cst, Dca, Rac, Tse and Vth investigated in this study), but for the other near half Al was toxic when the seeds were treated with its concentrations exceeding 1–2 mmol dm^−3^ (Abedi, Bartelheimer & Poschlod, 2013). Reduction of completion of germination (by c.a. 25%) due to increased availability of Al^3+^ (320 µmol dm^−3^) was also observed in *Ricinus communis* L. (Alves Silva et al., 2014) as well as in *Eugenia dysenterica* DC. (200–800 µmol Al^3+^ ⋅dm^−3^; reduction by 30%; Rodrigues et al., 2019). On the other hand, *L. sativa* is known to complete germination in the presence of Al within the range of c.a. 2–800 µmol Al^3+^ ⋅ dm^−3^, but concentrations higher that 2 µmol Al^3+^ ⋅ dm^−3^ slow down this process (Silva & Matos, 2016). Decelerated germination was also observed in *E. dysenterica* subjected to 600–800 µmol Al^3+^ ⋅ dm^−3^ (Rodrigues et al., 2019). Similar mode of action was also noted in this study, but the thresholds for the observed effect were species-specific. For example, undeniable basophilic species (Aam, Pgr and Sge) together with Dde (acidophilic species) were susceptible to Al toxicity even at its low concentrations, whereas the reduction of completion of germination for some other species was observed in 1 mmol Al^3+^ ⋅ dm^−3^ (Bof, Hpi, Pme and Pre) and in 10 mmol Al^3+^ ⋅ dm^−3^ (Evu, Gcr, Hra and Vte). It suggests that Al can be a barrier preventing establishment of basophiles on non-alkaline soils, whilst for acidophiles it probably contributes to the formation of a mosaic structure of vegetation. It must be noted however that slight acidification events in alkaline xerothermic grasslands probably do not lead to strong Al-driven shifts in germination-dependent revegetation, as basophilic species are able to complete germination efficiently under low Al concentrations. Furthermore, the basophiles seemed not to be more susceptible to Al toxicity than the tested acidophiles (hypothesis 3). It is known that the acquisition of Al is connected with generation of reactive oxygen species leading to oxidative stress and cellular damages (Šimonovičová et al., 2004; Bothe, 2015). It can be hypothesized that these processes occur also in a growing embryo when seeds encounter high amounts of freely available Al during imbibition. However, there are also some suggestions (Silva & Matos, 2016) that morpho-anatomical and chemical traits of a seed coat contribute to Al resistance at the germination stage, probably because of limited penetration of this element due to chemical constituents of this structure (most notably phenolic compounds that are able to complex Al^3+^ into insoluble forms; Moïse et al., 2005; Zhang et al., 2016). Alternatively, germinating seeds of tolerant species are able to withstand Al-caused toxicity on account of efficient stress coping mechanisms that reduce ion-specific toxicity and oxidative stress (resulting in fast compensation of damages). As many species in our investigation completed germination under high concentrations of Al, their tolerance buffer for completion of germination is wider than it could be predicted from their centers of abundance (Abedi, Bartelheimer & Poschlod, 2013). However, further studies are needed to determine if the tolerance level at the germination stage matches that at further ontogenetic stages, up to maturity (including reproduction). It can be predicted that if the tolerance buffer to Al toxicity during germination of a given species is greater than those during further developmental stages (*e.g.*, growth of seedlings), depletion of seed banks is very plausible, which was proposed for other environmental stimuli, *e.g.*, nitrogen (Bird & Choi, 2017).

Interestingly, some species may even experience positive effects of increased availability of Al, *e.g.*, remarkably acidophilic grass, *Corynephorus canescens* L. (most markedly at 0.01 mmol Al^3+^ ⋅ dm^−3^, but possibly up to 2 mmol Al^3+^ ⋅ dm^−3^; Abedi, Bartelheimer & Poschlod, 2013). Similar slight stimulation was observed in Hpe and Csc (0.01 and 10 mmol Al^3+^ ⋅ dm^−3^, respectively) investigated in this study. Enhanced completion of germination may be in this case the result of an increased pro-oxidative state due to Al-dependent stimuli (Šimonovičová et al., 2004), as reactive oxygen species (most notably hydrogen peroxide) are known to trigger the release from dormancy and accelerate remobilization of resources and endosperm weakening (Wojtyla et al., 2016). It can be proposed that such stimulation plays a role in detection of optimal conditions for growth and development in some Al-tolerant specialists (in this case Hpe and Csc).

Finally, the presented study showed that flora from both acidic dry and alkaline xerothermic grasslands was diverse in its germination strategies. Hierarchical clustering analysis indicated that there was no homogenous reaction to the tested conditions within each group. Furthermore, seed size and estimated preference for soil acidity should not be used as common delimiters allowing sharp segregation of germination strategies of acidophilic and basophilic plants (in terms of influence of edaphic conditions, *e.g.*, pH, Al, Fe and Mn). Moreover, seed weight did not correlate with the ability of seeds to complete germination under increased availability of the tested metals or changing acidity (with sparse exceptions). This suggests the existence of some more complicated species-specific structural and/or physiological adaptations in seeds that are probably not yet well recognized to be the traits associated with tolerance to chemical stimuli.

## Conclusion

Overall, the present study showed a diversity of germination strategies in plants from acidic dry as well as alkaline xerothermic grasslands. Tolerances to pH and availability of Fe, Mn and Al are other traits contributing to floristic diversity of a given plant community due to the formation of microhabitats. This seems to promote mosaic structure of the studied plant communities; however; in our opinion pH and the extremely high availability of Al are major factors, while availability of Fe and Mn is probably of secondary importance. The studied edaphic factors seem to filter plant establishment on remarkably acidic and alkaline soils and might be among the driving forces of evolution of plant germination strategies. As specialization of germination requirements in the studied species was found, changes in soil pH and availability of Al, Fe and Mn should be taken into consideration in further studies on biology of dry acidic, and xerothermic alkaline grasslands. It can be expected that spatial and temporal changes in local vegetation have an impact on germination-based revegetation in the studied types of communities. Thus, we believe that ecology of acidic and alkaline grasslands should be co-investigated with soil chemistry. Such studies cannot only deepen understanding of species diversity and natural processes in these kinds of communities (*e.g.*, succession), but also can improve management, protection and ecological restoration of grasslands in a worldwide scale. Furthermore, both Fe-HBED and Mn-HBED exerted similar effects on the ability of seeds to complete germination when compared to ionic forms of these metals. This suggests that both chelates are not detrimental to early ontogenetic stages of plants when they are reasonably used.

## Supplemental Information

10.7717/peerj.13255/supp-1 Supplemental Information 1 Acidity of the tested Fe-, Mn- and Al-containing solutions presented as pH values The pH value of water used for control variants and for preparation of the tested solutions was 5.81.

10.7717/peerj.13255/supp-2 Supplemental Information 2 Results of two-way ANOVA (*F* values and significance) showing influence of the studied factors (type and dose of the tested compounds) and their interactions on final seed germination percentage (FGP) and index of germination velocity (IGV) of the st^*a*^ FeCl_3_ or Fe-HBED/MnCl_2_ or Mn-HBED; ^*b*^ 5 or 25 µmol dm−3 Differences considered as statistically significant (*p* < 0.05) were bolded.

10.7717/peerj.13255/supp-3 Supplemental Information 3 Comparison of effects of isomolar solutions of tested metals (Me) on the ability of the seeds to complete germination Data are presented as FGPs ratio between values recorded in Experiment 2 (effect of chelated and ionic Fe) and values recorded in Experiment 3 (effect of chelated and ionic Mn), where Me means Fe and Mn. Comparison was conducted using raw FGPs values (two-way ANOVA followed by Bonferroni’s post-hoc test; *n* = 4). Differences considered as statistically significant (*p* < 0.05) were bolded.

10.7717/peerj.13255/supp-4 Supplemental Information 4 Results of three-way ANOVA (*F* values and significance) showing influence of the studied factors (metal, type and dose of the tested compounds) and their interactions on final seed germination percentage (FGP) and index of germination velocity (IGV) ^*a*^Fe or Mn; ^*b*^MeCl_*X*_ or Me-HBED; ^*c*^ 5 or 25 µmol dm^−3^ Differences considered as statistically significant (*p* < 0.05) were bolded.

10.7717/peerj.13255/supp-5 Supplemental Information 5 Coefficients of correlation between relative response of seeds to the tested conditions and seed weight calculated using Speraman’s sum rank test Analysis was conducted using data from Experiments 1–4 for all tested species and for divided groups of acidophilous and basiphilous species. For each species, the data were normalized to FGP values recorded at pH = 7 (Experiment 1) or control (null concentration) conditions (Experiments 2–4) and weight of seeds were took from presented investigation (Table 1). Data is presented as coefficients with respective p values (showed in parentheses). Significant correlations were bolded (*p* < 0.05).

10.7717/peerj.13255/supp-6 Supplemental Information 6 LC-MS/MS spectra (A, B, E, F) , FTIR spectra (C, G) and UV–VIS spectra (D, H) of Fe-HBED and Mn-HBED

10.7717/peerj.13255/supp-7 Supplemental Information 7 Raw data of the germination-related traits The raw data were used for all statistical analyses.

## Additional Information and Declarations

### Data Availability

The following information was supplied regarding data availability: The raw measurements (values of final germination percentage and index of germination velocity) are available in the Supplementary File.
