# Supplementary material for: Acidity and availability of aluminum, iron and manganese as factors affecting germination in European acidic dry and alkaline xerothermic grasslands
Source: PeerJ. 2022 Apr 28;10:e13255. doi: 10.7717/peerj.13255 (PMC9057293; doi:10.7717/peerj.13255)
Supplement: Supplemental Information 1 — The pH value of water used for control variants and for preparation of the tested solutions was 5.81. [file peerj-10-13255-s001.docx]

**Supplemental Table 1. Acidity of the tested Fe-, Mn- and Al-containing solutions presented as pH values.** The pH value of water used for control variants and for preparation of the tested solutions was 5.81.

| Concentration of tested solution | | pH value |
| --- | --- | --- |
| FeCl_3_ [µmol · dm^−3^] | 5 | 5.10 |
|  | 25 | 4.37 |
| Fe-HBED [µmol · dm^−3^] | 5 | 5.75 |
|  | 25 | 5.74 |
| MnCl_2_ [µmol · dm^−3^] | 5 | 5.40 |
|  | 25 | 5.31 |
| Mn-HBED [µmol · dm^−3^] | 5 | 5.82 |
|  | 25 | 5.99 |
| AlCl_3_ [mmol · dm^−3^] | 0.01 | 5.38 |
|  | 0.10 | 4.41 |
|  | 1.00 | 3.89 |
|  | 10.00 | 3.69 |
