# Supplementary material for: Acidity and availability of aluminum, iron and manganese as factors affecting germination in European acidic dry and alkaline xerothermic grasslands
Source: PeerJ. 2022 Apr 28;10:e13255. doi: 10.7717/peerj.13255 (PMC9057293; doi:10.7717/peerj.13255)
Supplement: Supplemental Information 2 — Differences considered as statistically significant (p < 0.05) were bolded. [file peerj-10-13255-s002.docx]

**Supplemental Table 2. Results of two-way ANOVA (*F* values and significance) showing influence of the studied factors (type and dose of the tested compounds) and their interactions on final seed germination percentage (FGP) and index of germination velocity (IGV) of the studied species.** ^a^FeCl_3_ or Fe-HBED/MnCl_2_ or Mn-HBED; ^b^5 or 25 µmol · dm^−3­­^. Differences considered as statistically significant (p < 0.05) were bolded.

| Species | *F* values and significance | | | | | |
| --- | --- | --- | --- | --- | --- | --- |
|  | Experiment 2 | | | Experiment 3 | | |
|  | Fe type^a^  (FeT, *df* = 1) | Fe dose^b^  (FeD, *df* = 1) | FeT x FeD  (*df* = 3) | Mn type^a^  (MnT, *df* = 1) | Mn dose^b^  (MnD, *df* = 1) | MnT x MnD  (*df* = 3) |
|  | FGP | | | | | |
| Amo | 272.3 (**<0.001**) | 21.1 (**<0.001**) | 45.7 (**<0.001**) | 1.0 (0.343) | 53.3 (**<0.001**) | 1.9 (0.192) |
| Aam | 22.1 (**<0.001**) | 22.1 (**<0.001**) | 2.5 (0.143) | 7.4 (**0.019**) | 0.5 (0.510) | 0.5 (0.510) |
| Bof | 0.7 (0.427) | 0.7 (0.427) | 0.2 (0.631) | 21.4 (**<0.001**) | 0.2 (0.682) | 0.2 (0.682) |
| Csc | 1.0 (0.327) | 12.8 (**0.004**) | 0.3 (0.619) | 4.4 (0.058) | 14.3 (**0.003**) | 0.2 (0.682) |
| Cst | 0.2 (0.663) | 0.2 (0.663) | 0.2 (0.663) | 1.0 (0.337) | 0.1 (0.745) | 0.1 (0.745) |
| Dca | 0.2 (0.646) | 5.6 (**0.036**) | 0.9 (0.364) | 0.2 (0.657) | 0.2 (0.657) | 0.8 (0.381) |
| Dde | 4.3 (0.061) | 64.5 (**<0.001**) | 44.3 (**<0.001**) | 34.4 (**<0.001**) | 0.4 (0.545) | 0.4 (0.545) |
| Evu | 22.1 (**<0.001**) | 3.0 (0.109) | 0.1 (0.809) | 0.2 (0.648) | 0.1 (0.878) | 0.6 (0.450) |
| Gcr | 82.3 (**<0.001**) | 0.1 (0.828) | 0.1 (0.828) | 1.3 (0.274) | 2.0 (0.177) | 3.0 (0.111) |
| Hpi | 13.8 (**0.003**) | 1.2 (0.297) | 1.2 (0.297) | 0.1 (0.721) | 0.1 (1.000) | 0.5 (0.479) |
| Hpe | 0.8 (0.396) | 26.0 (**<0.001**) | 22.5 (**<0.001**) | 0.1 (0.847) | 1.0 (0.343) | 0.1 (0.847) |
| Hra | 22.0 (**<0.001**) | 65.6 (**<0.001**) | 18.2 (**0.001**) | 1.1 (0.306) | 7.1 (**0.020**) | 0.3 (0.603) |
| Pme | 3.2 (0.099) | 4.6 (0.053) | 3.2 (0.099) | 32.7 (**<0.001**) | 0.2 (0.668) | 0.1 (1.000) |
| Pre | 19.2 (**<0.001**) | 0.1 (0.721) | 0.5 (0.479) | 0.5 (0.512) | 0.1 (0.825) | 0.1 (0.825) |
| Pgr | 5.2 (**0.041**) | 0.8 (0.396) | 0.3 (0.607) | 1.3 (0.284) | 2.2 (0.161) | 0.1 (0.715) |
| Rac | 9.3 (**0.010**) | 2.6 (0.132) | 0.8 (0.387) | 17.0 (**0.001**) | 14.2 (**0.003**) | 17.0 (**0.001**) |
| Sge | 1.5 (0.242) | 37.9 (**<0.001**) | 0.3 (0.607) | 1.3 (0.272) | 0.2 (0.631) | 0.2 (0.631) |
| Tse | 0.9 (0.358) | 0.1 (1.000) | 0.1 (0.755) | 2.0 (0.178) | 1.1 (0.305) | 0.1 (1.000) |
| Vth | 0.1 (0.801) | 15.0 (**0.002**) | 0.6 (0.454) | 0.1 (0.773) | 0.1 (0.773) | 0.3 (0.566) |
| Vte | 4.1 (0.065) | 8.6 (**0.013**) | 1.3 (0.282) | 0.4 (0.565) | 0.4 (0.565) | 0.1 (0.847) |
|  | IGV | | | | | |
| Amo | 309.5 (**<0.001**) | 24.1 (**<0.001**) | 58.5 (**<0.001**) | 1.3 (0.269) | 53.8 (**<0.001**) | 2.5 (0.143) |
| Aam | 41.3 (**<0.001**) | 15.5 (**0.002**) | 1.0 (0.344) | 16.4 (**0.002**) | 1.1 (0.314) | 0.7 (0.404) |
| Bof | 1.2 (0.296) | 1.1 (0.312) | 0.1 (0.915) | 23.2 (**<0.001**) | 0.7 (0.422) | 0.1 (1.000) |
| Csc | 0.1 (0.724) | 15.4 (**0.002**) | 0.1 (0.782) | 4.2 (0.063) | 13.5 (**0.003**) | 0.2 (0.700) |
| Cst | 7.1 (**0.020**) | 1.3 (0.282) | 1.2 (0.303) | 1.3 (0.286) | 0.6 (0.470) | 0.3 (0.576) |
| Dca | 0.3 (0.581) | 5.8 (**0.033**) | 0.7 (0.430) | 0.8 (0.399) | 0.5 (0.474) | 1.0 (0.346) |
| Dde | 3.7 (0.079) | 70.1 (**<0.001**) | 43.3 (**<0.001**) | 35.5 (**<0.001**) | 0.2 (0.644) | 1.0 (0.339) |
| Evu | 28.7 (**<0.001**) | 4.0 (0.069) | 0.5 (0.492) | 0.2 (0.650) | 0.1 (0.723) | 0.6 (0.446) |
| Gcr | 75.1 (**<0.001**) | 0.1 (0.787) | 2.8 (0.117) | 1.6 (0.233) | 3.0 (0.109) | 5.1 (**0.043**) |
| Hpi | 11.3 (**0.006**) | 4.0 (0.068) | 1.6 (0.232) | 0.2 (0.636) | 0.1 (0.834) | 0.1 (0.811) |
| Hpe | 19.9 (**<0.001**) | 16.6 (**0.002**) | 32.1 (**<0.001**) | 3.2 (0.101) | 3.4 (0.090) | 1.0 (0.345) |
| Hra | 33.3 (**<0.001**) | 74.3 (**<0.001**) | 20.6 (**<0.001**) | 0.9 (0.358) | 6.0 (**0.030**) | 0.5 (0.485) |
| Pme | 2.9 (0.114) | 6.2 (**0.029**) | 3.3 (0.095) | 30.5 (**<0.001**) | 0.8 (0.391) | 0.3 (0.620) |
| Pre | 19.5 (**<0.001**) | 0.1 (0.938) | 0.4 (0.556) | 0.3 (0.625) | 0.1 (0.765) | 0.1 (0.848) |
| Pgr | 14.6 (**0.002**) | 0.7 (0.435) | 0.3 (0.586) | 3.1 (0.102) | 2.2 (0.164) | 0.3 (0.592) |
| Rac | 13.2 (**0.003**) | 5.6 (**0.036**) | 1.8 (0.210) | 15.1 (**0.002**) | 10.0 (**0.008**) | 19.7 (**<0.001**) |
| Sge | 0.6 (0.444) | 34.2 (**<0.001**) | 0.8 (0.395) | 1.4 (0.253) | 0.1 (0.755) | 0.5 (0.500) |
| Tse | 1.0 (0.339) | 0.1 (0.914) | 0.1 (0.929) | 2.3 (0.154) | 0.8 (0.377) | 0.1 (0.966) |
| Vth | 0.9 (0.371) | 20.0 (**<0.001**) | 2.2 (0.162) | 0.4 (0.539) | 0.3 (0.572) | 0.4 (0.518) |
| Vte | 4.3 (0.060) | 7.9 (**0.016**) | 3.7 (0.079) | 0.1 (0.732) | 0.5 (0.499) | 0.1 (0.827) |
