# Supplementary material for: Acidity and availability of aluminum, iron and manganese as factors affecting germination in European acidic dry and alkaline xerothermic grasslands
Source: PeerJ. 2022 Apr 28;10:e13255. doi: 10.7717/peerj.13255 (PMC9057293; doi:10.7717/peerj.13255)
Supplement: Supplemental Information 3 — Data are presented as FGPs ratio between values recorded in Experiment 2 (effect of chelated and ionic Fe) and values recorded in Experiment 3 (effect of chelated and ionic Mn), where Me means Fe and Mn. Comparison was conducted using raw FGPs values (two-way ANOVA followed by Bonferroni’s post-hoc test; n = 4). Differences considered as statistically significant (p < 0.05) were bolded. [file peerj-10-13255-s003.docx]

**Supplemental Table 3. Comparison of effects of isomolar solutions of tested metals (Me) on the ability of the seeds to complete germination.** Data are presented as FGPs ratio between values recorded in Experiment 2 (effect of chelated and ionic Fe) and values recorded in Experiment 3 (effect of chelated and ionic Mn), where Me means Fe and Mn. Comparison was conducted using raw FGPs values (two-way ANOVA followed by Bonferroni’s post-hoc test; n = 4). Differences considered as statistically significant (p<0.05) were bolded.

| Species | MeCl_X_ | | Me-HBED | |
| --- | --- | --- | --- | --- |
|  | 5 µmol · dm^−3^ | 25 µmol · dm^−3^ | 5 µmol · dm^−3^ | 25 µmol · dm^−3^ |
| Amo | 1.317 (**<0.001**) | 2.122 (**<0.001**) | 1.048 (1.000) | 0.936 (1.000) |
| Aam | 1.429 (**0.002**) | 1.263 (0.108) | 1.375 (**0.001**) | 1.250 (**0.023**) |
| Bof | 1.385 (**0.025**) | 1.486 (**0.007**) | 1.020 (1.000) | 1.102 (1.000) |
| Csc | 1.783 (**0.001**) | 2.692 (**<0.001**) | 1.630 (**<0.001**) | 1.895 (**<0.001**) |
| Cst | 1.010 (1.000) | 1.010 (1.000) | 1.000 (1.000) | 1.000 (1.000) |
| Dca | 0.968 (1.000) | 0.884 (**0.037**) | 0.926 (0.159) | 0.924 (0.159) |
| Dde | 1.000 (1.000) | 0.522 (**<0.001**) | 1.043 (1.000) | 0.900 (0.962) |
| Evu | 1.250 (**0.044**) | 1.130 (0.653) | 1.038 (1.000) | 1.040 (1.000) |
| Gcr | 0.863 (0.240) | 0.852 (0.164) | 1.098 (0.223) | 1.254 (**0.001**) |
| Hpi | 0.923 (0.456) | 0.944 (1.000) | 0.867 (**0.032**) | 0.793 (**0.001**) |
| Hpe | 1.123 (0.565) | 1.145 (0.360) | 1.293 (**0.004**) | 0.855 (0.330) |
| Hra | 1.125 (0.479) | 0.595 (**<0.001**) | 0.673 (**<0.001**) | 0.533 (**<0.001**) |
| Pme | 1.179 (0.115) | 1.182 (0.115) | 0.957 (1.000) | 0.809 (**0.018**) |
| Pre | 1.068 (1.000) | 1.081 (0.693) | 0.945 (1.000) | 0.917 (0.680) |
| Pgr | 0.815 (**0.009**) | 0.816 (**0.015**) | 0.909 (0279) | 0.929 (0.732) |
| Rac | 1.000 (1.000) | 1.271 (**0.032**) | 0.866 (**0.029**) | 0.831 (**0.005**) |
| Sge | 1.145 (0.247) | 0.932 (1.000) | 1.188 (0.107) | 0.891 (0.815) |
| Tse | 0.900 (0.843) | 0.925 (1.000) | 0.985 (1.000) | 1.048 (1.000) |
| Vth | 1.022 (1.000) | 0.914 (0.367) | 1.000 (1.000) | 0.967 (1.000) |
| Vte | 0.978 (1.000) | 0.868 (**0.021**) | 0.989 (1.000) | 0.925 (0.378) |
