# Supplementary material for: Acidity and availability of aluminum, iron and manganese as factors affecting germination in European acidic dry and alkaline xerothermic grasslands
Source: PeerJ. 2022 Apr 28;10:e13255. doi: 10.7717/peerj.13255 (PMC9057293; doi:10.7717/peerj.13255)
Supplement: Supplemental Information 5 — Analysis was conducted using data from Experiments 1–4 for all tested species and for divided groups of acidophilous and basiphilous species. For each species, the data were normalized to FGP values recorded at pH = 7 (Experiment 1) or control (null concentration) conditions (Experiments 2–4) and weight of seeds were took from presented investigation (Table 1). Data is presented as coefficients with respective p values (showed in parentheses). Significant correlations were bolded (p < 0.05). [file peerj-10-13255-s005.docx]

**Supplemental Table 5. Coefficients of correlation between relative response of seeds to the tested conditions and seed weight calculated using Speraman’s sum rank test.** Analysis was conducted using data from Experiments 1-4 for all tested species and for divided groups of acidophilous and basiphilous species. For each species, the data were normalized to FGP values recorded at pH = 7 (Experiment 1) or control (null concentration) conditions (Experiments 2-4) and weight of seeds were took from presented investigation (Table 1). Data is presented as coefficients with respective p values (showed in parentheses). Significant correlations were bolded (p < 0.05).

| Conditions | Acidophilous species  (n = 10) | Basophilous species  (n = 10) | All species  (n = 20) |
| --- | --- | --- | --- |
|  |  | FGP |  |
| Acidity (pH) |  |  |  |
| 5.0 | -0,479 (0.162) | -0.358 (0.310) | -0.430 (0.058) |
| 6.0 | -0.309 (0.385) | -0.030 (0.934) | -0.200 (0.398) |
| 8.0 | 0.127 (0.726) | 0.055 (0.881) | 0.023 (0.925) |
| Iron (Fe) |  |  |  |
| 5 µmol · dm^−3^ FeCl_3_ | 0.139 (0.701) | 0.248 (0.489) | 0.090 (0.705) |
| 25 µmol · dm^−3^ FeCl_3_ | 0.333 (0.347) | -0.261 (0.467) | 0.059 (0.803) |
| 5 µmol · dm^−3^ Fe-HBED | -0.127 (0.726) | 0.024 (0.947) | -0.050 (0.835) |
| 25 µmol · dm^−3^ Fe-HBED | 0.164 (0.651) | -0.224 (0.533) | 0.051 (0.830) |
| Manganese (Mn) |  |  |  |
| 5 µmol · dm^−3^ MnCl_2_ | -0.164 (0.651) | -0.491 (0.150) | **-0.447 (0.048)** |
| 25 µmol · dm^−3^ MnCl_2_ | -0.309 (0.385) | -0.539 (0.108) | -0.423 (0.063) |
| 5 µmol · dm^−3^ Mn-HBED | 0.297 (0.405) | -0.552 (0.098) | -0.206 (0.383) |
| 25 µmol · dm^−3^ Mn-HBED | 0.152 (0.675) | -0.285 (0.425) | -0.126 (0.598) |
| Aluminum (Al) |  |  |  |
| 0.01 mmol · dm^−3^ | -0.624 (0.054) | -0.505 (0.137) | **-0.510 (0.022)** |
| 0.10 mmol · dm^−3^ | -0.188 (0.603) | -0.212 (0.556) | -0.173 (0.466) |
| 1.00 mmol · dm^−3^ | 0.454 (0.187) | -0.176 (0.627) | 0.066 (0.782) |
| 10.00 mmol · dm^−3^ | 0.345 (0.328) | -0.309 (0.385) | -0.063 (0.791) |
|  |  | IGV |  |
| Acidity (pH) |  |  |  |
| 5.0 | -0.430 (0.214) | -0.321 (0.365) | -0.415 (0.069) |
| 6.0 | -0.345 (0.328) | -0.067 (0.855) | -0.192 (0.416) |
| 8.0 | 0.261 (0.467) | 0.103 (0.777) | 0.062 (0.796) |
| Iron (Fe) |  |  |  |
| 5 µmol · dm^−3^ FeCl_3_ | -0.309 (0.385) | 0.406 (0.244) | -0.135 (0.571) |
| 25 µmol · dm^−3^ FeCl_3_ | -0.042 (0.907) | -0.285 (0.425) | -0.102 (0.668) |
| 5 µmol · dm^−3^ Fe-HBED | -0.188 (0.603) | 0.285 (0.425) | -0.030 (0.900) |
| 25 µmol · dm^−3^ Fe-HBED | 0.176 (0.626) | -0.248 (0.489) | 0.076 (0.750) |
| Manganese (Mn) |  |  |  |
| 5 µmol · dm^−3^ MnCl_2_ | -0.127 (0.726) | 0.182 (0.614) | -0.363 (0.115) |
| 25 µmol · dm^−3^ MnCl_2_ | -0.152 (0.676) | -0.134 (0.713) | -0.355 (0.125) |
| 5 µmol · dm^−3^ Mn-HBED | 0.333 (0.347) | 0.115 (0.751) | -0.062 (0.796) |
| 25 µmol · dm^−3^ Mn-HBED | 0.309 (0.385) | 0.358 (0.310) | 0.002 (0.995) |
| Aluminum (Al) |  |  |  |
| 0.01 mmol · dm^−3^ | **-0.648 (0.043)** | -0.382 (0.276) | **-0.503 (0.024)** |
| 0.10 mmol · dm^−3^ | -0.103 (0.777) | -0.152 (0.676) | -0.161 (0.498) |
| 1.00 mmol · dm^−3^ | 0.527 (0.117) | -0.079 (0.829) | 0.191 (0.420) |
| 10.00 mmol · dm^−3^ | 0.418 (0.229) | -0.297 (0.405) | 0.017 (0.945) |
