# Supplementary material for: Acidity and availability of aluminum, iron and manganese as factors affecting germination in European acidic dry and alkaline xerothermic grasslands
Source: PeerJ. 2022 Apr 28;10:e13255. doi: 10.7717/peerj.13255 (PMC9057293; doi:10.7717/peerj.13255)
Supplement: Supplemental Information 6 [file peerj-10-13255-s006.pdf]

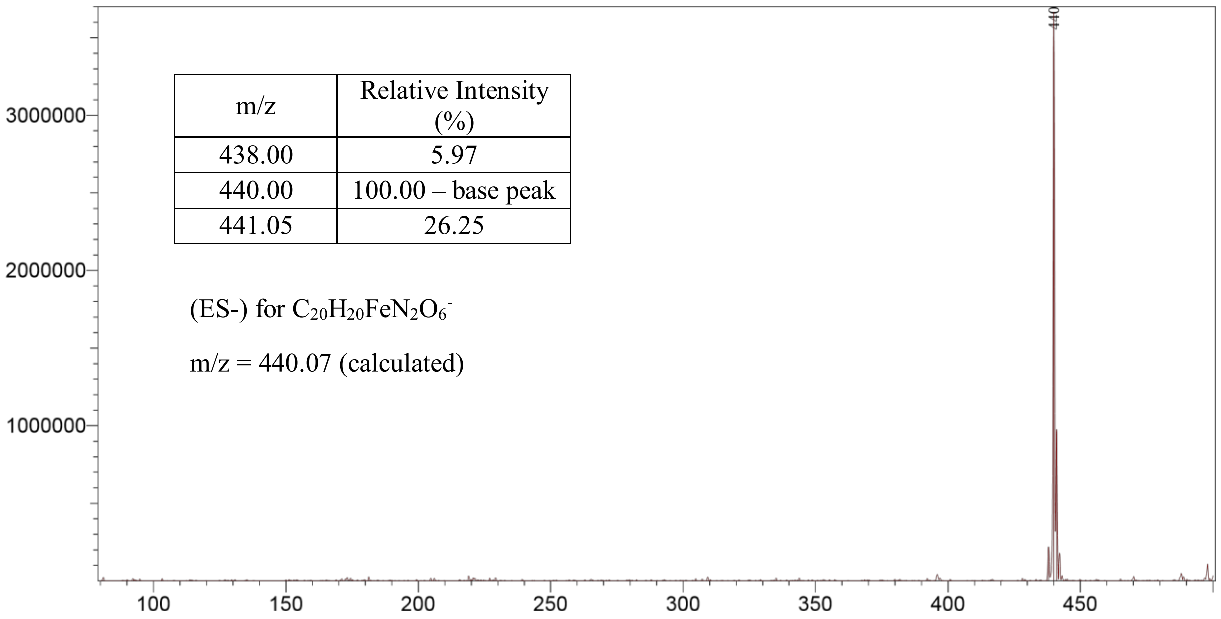

A. LC-MS/MS spectrum (ES-) of *N*,*N*'-di(2-hydroxybenzyl)ethylenediamine-*N*,*N*'-diacetic acid iron(III) sodium salt, Fe-HBED.

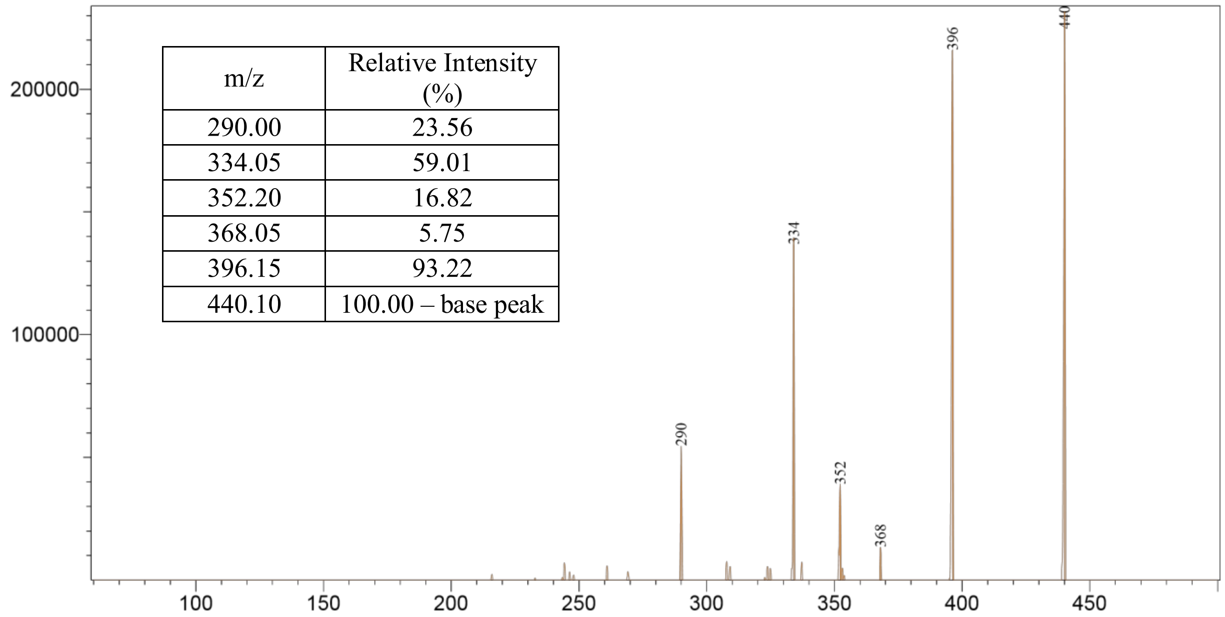

B. LC-MS/MS spectrum (ES-) of *N*,*N*'-di(2-hydroxybenzyl)ethylenediamine-*N*,*N*'-diacetic acid iron(III) sodium salt, Fe-HBED – base peak (m/z 440.10) fragmentation spectrum.

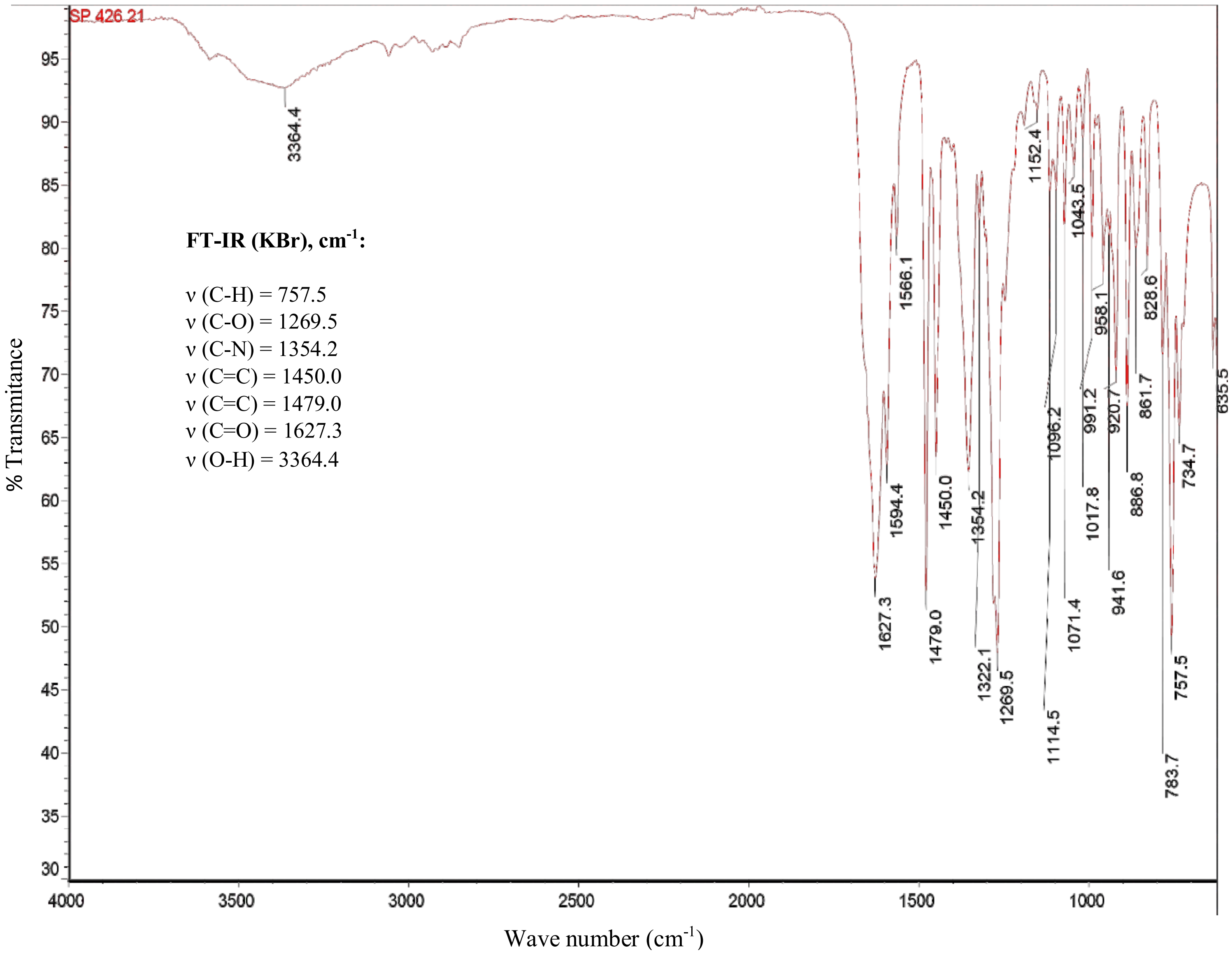

C. FT-IR spectrum of *N*,*N*'-di(2-hydroxybenzyl)ethylenediamine-*N*,*N*'-diacetic acid iron(III) sodium salt, Fe-HBED.

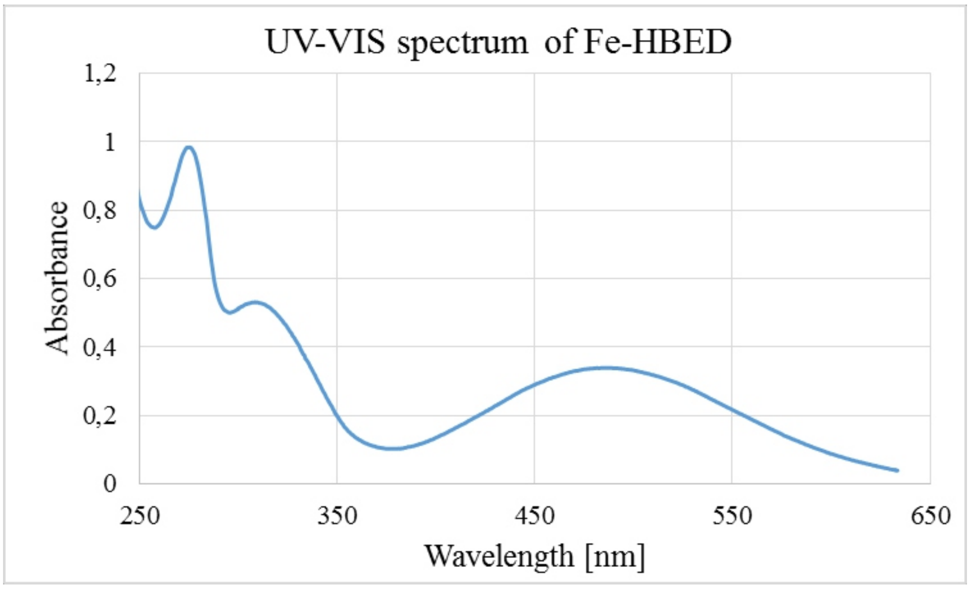

$\lambda_{max1} = 274.0$ nm; $\lambda_{max2} = 309.0$ nm; $\lambda_{max3} = 491.5$ nm;

$\varepsilon = 357.48\ dm^3 \cdot mol^{-1} \cdot cm^{-1}$

D. UV-VIS spectrum of *N*,*N*'-di(2-hydroxybenzyl)ethylenediamine-*N*,*N*'-diacetic acid iron(III) sodium salt, Fe-HBED.

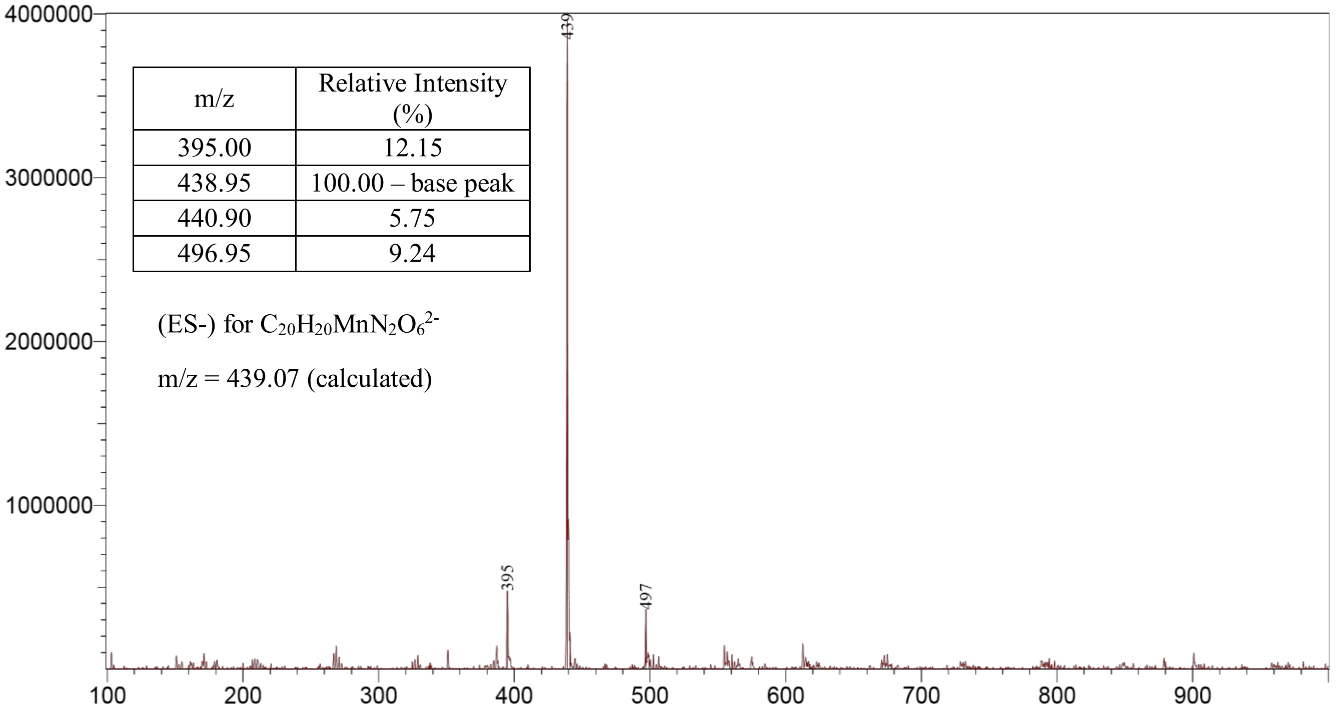

m/z	Relative Intensity (%)
395.00	12.15
438.95	100.00 – base peak
440.90	5.75
496.95	9.24

E. LC-MS/MS spectrum (ES-) of *N*,*N*'-di(2-hydroxybenzyl)ethylenediamine-*N*,*N*'-diacetic acid manganese(II) sodium salt, Mn-HBED.

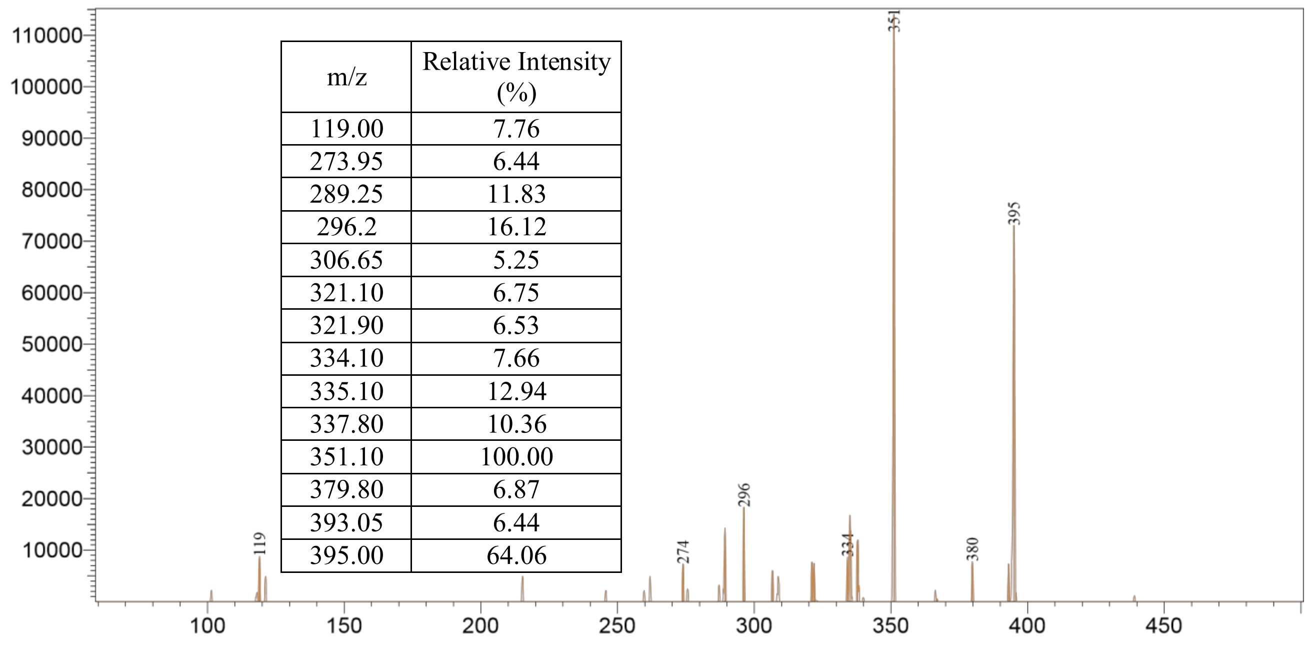

m/z	Relative Intensity (%)
119.00	7.76
273.95	6.44
289.25	11.83
296.2	16.12
306.65	5.25
321.10	6.75
321.90	6.53
334.10	7.66
335.10	12.94
337.80	10.36
351.10	100.00
379.80	6.87
393.05	6.44
395.00	64.06

F. LC-MS/MS spectrum (ES-) of *N*,*N*'-di(2-hydroxybenzyl)ethylenediamine-*N*,*N*'-diacetic acid manganese(II) sodium salt, Mn-HBED – base peak (m/z 438.95) fragmentation spectrum.

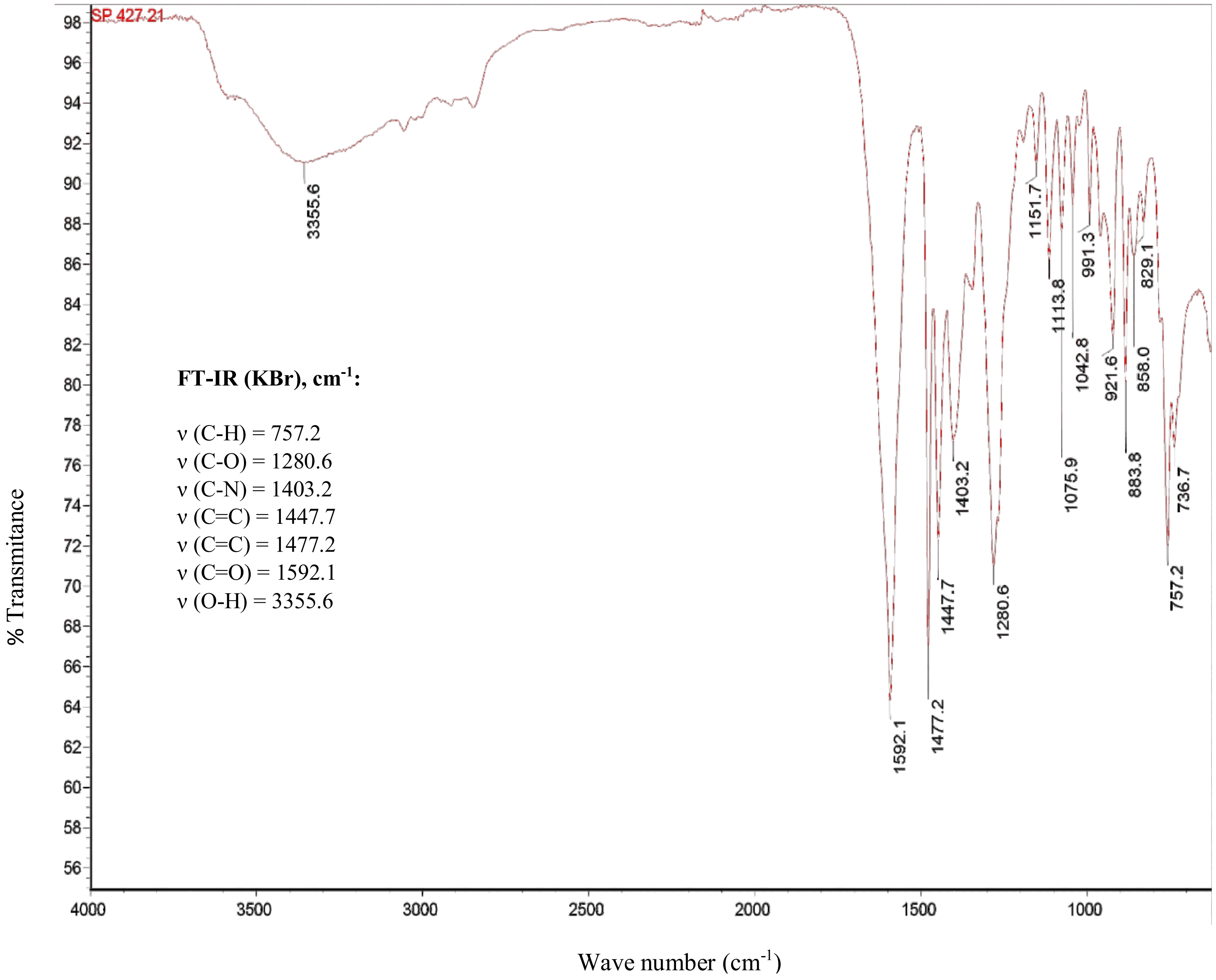

G. FT-IR spectrum of *N*,*N*'-di(2-hydroxybenzyl)ethylenediamine-*N*,*N*'-diacetic acid manganese(II) sodium salt, Mn-HBED.

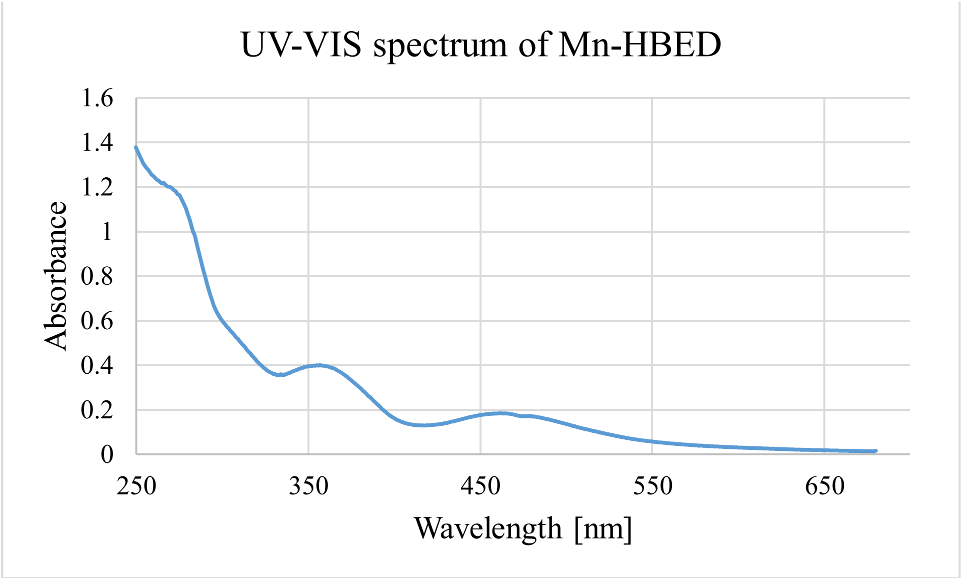

λ_{max1} = 269.0 nm; λ_{max2} = 355.0 nm; λ_{max3} = 463.5 nm;

$\varepsilon = 675.01\ dm^3 \cdot mol^{-1} \cdot cm^{-1}$

H. UV-VIS spectrum of *N*,*N*'-di(2-hydroxybenzyl)ethylenediamine-*N*,*N*'-diacetic acid manganese(II) sodium salt, Mn-HBED.

Supplementary Figure S1. LC-MS/MS spectra (A, B, E, F) , FTIR spectra (C, G) and UV-VIS spectra (D, H) of Fe-HBED and Mn-HBED.
